# Sphingosine kinase 2 deficiency impairs VLDL secretion by inhibiting mTORC2 phosphorylation and activating chaperone-mediated autophagy

**DOI:** 10.1038/s41418-025-01507-6

**Published:** 2025-04-08

**Authors:** Shuangshuang Zhang, Gaoxiang Li, Lianping He, Fei Wang, Mengru Gao, Tianliang Dai, Yushuang Su, Luyan Li, Ying Cao, Minghua Zheng, Liang Chen, Jun Cao, Hong Zhou

**Affiliations:** 1 School of Life Sciences, Anhui Medical University, Hefei, 230032 Anhui China; 2 Department of Immunology, Nanjing Medical University, Nanjing, 211166 China; 3 Clinical Pathology Center, the First Affiliated Hospital of Anhui Medical University, Hefei, 230012 Anhui China; 4 Anhui Public Health Clinical Center, Hefei, 230012 Anhui China; 5 NAFLD Research Center, Department of Hepatology, the First Affiliated Hospital of Wenzhou Medical University, Wenzhou, 325000 China; 6 Key Laboratory of Diagnosis and Treatment for the Development of Chronic Liver Disease in Zhejiang Province, Wenzhou, 325000 China

**Keywords:** Autophagy, Proteomics

## Abstract

Hepatic very low-density lipoprotein (VLDL) is essential for maintaining lipid metabolism in the liver. Sphingosine kinases (SphKs) are essential rate-limiting enzymes that catalyze sphingosine phosphorylation to Sphingosine-1-phosphate (S1P). SphKs exist as two isoforms, SphK1 and SphK2, both highly expressed in the liver. SphK1 plays a critical role in regulating hepatic inflammation and drug metabolism. This study aimed to determine whether SphK2 regulates hepatic lipid metabolism, particularly VLDL secretion. Immunohistochemical staining revealed decreased SphK2 protein levels within regions proximal to hepatic lipid accumulation in individuals diagnosed with metabolic dysfunction-associated steatotic liver disease (MASLD). *Sphk2*^*−/−*^ mice exhibited spontaneous hepatocyte lipid accumulation and reduced VLDL secretion. Proteomic analysis revealed that SphK2 deficiency impaired soluble N-ethylmaleimide-sensitive fusion attachment protein receptor (SNARE) complex interactions involved in vesicular transport and organelle membrane fusion. Furthermore, SphK2 deficiency results in accelerated degradation of the SEC22B, STX5A, and GS28 proteins via chaperone-mediated autophagy (CMA), impeding VLDL transport to the Golgi apparatus. MYH1485, a specific activator of mTOR, induces mTORC2 phosphorylation, thereby inhibiting the degradation of SNARE complexes by CMA and counteracting the lipid accumulation induced by SphK2 deficiency. Exogenous S1P supplementation markedly reversed the reduction in mTORC2 phosphorylation and suppressed CMA, thereby improving VLDL secretion. Our study elucidates an inventive regulatory mechanism by which SphK2 modulates CMA by activating mTORC2 phosphorylation, promoting VLDL secretion, and balancing lipid metabolism in the liver. These findings provide insights into SphK2 function and the underlying mechanisms involved in the regulation of VLDL secretion, which may facilitate MASLD treatment.

**Figure:**
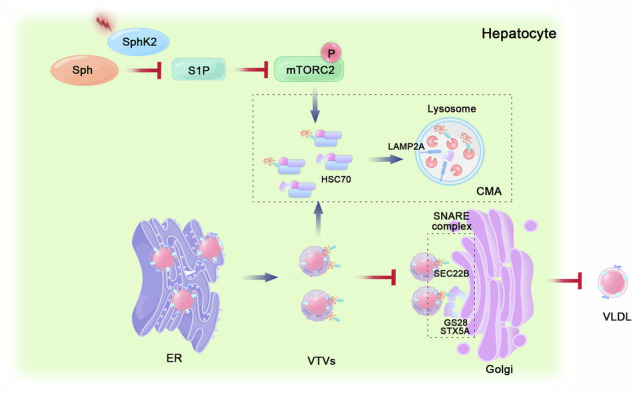
**Proposed model for the role of SphK2 in hepatic VLDL secretion**. In hepatocytes, the inhibition of SphK2 activity decreased S1P production, which subsequently downregulates the mTORC2 pathway. This process accelerates the degradation of the SNARE complex components STX5A, GS28, and SEC22B via CMA, which regulates the mutual recognition between VTVs and the Golgi apparatus, ultimately reducing VLDL secretion in hepatocytes.

**Proposed model for the role of SphK2 in hepatic VLDL secretion**. In hepatocytes, the inhibition of SphK2 activity decreased S1P production, which subsequently downregulates the mTORC2 pathway. This process accelerates the degradation of the SNARE complex components STX5A, GS28, and SEC22B via CMA, which regulates the mutual recognition between VTVs and the Golgi apparatus, ultimately reducing VLDL secretion in hepatocytes.

## Introduction

Metabolic dysfunction-associated steatotic liver disease (MASLD), characterized by the accumulation of lipids in the liver in association with metabolic syndrome, remains a major global health concern, often impacting individuals with concurrent ailments such as type 2 diabetes and dyslipidemia [1]. Hepatic steatosis is characterized by the accumulation of atypical and excessive lipids, specifically triglycerides (TGs) and cholesteryl esters, in the liver [2]. Lipid homeostasis in the liver is regulated by various processes, including de novo lipogenesis, fatty acid (FA) uptake, esterification, and oxidation, as well as glucose metabolism, particularly the secretion of very low-density lipoproteins (VLDLs) [3–5].

Upon synthesis within the endoplasmic reticulum lumen, VLDLs are transported to the *cis*-Golgi apparatus and subsequently secreted into the circulatory system [6]. Disruptions to this process may lead to anomalous hepatic lipid accumulation, further progressing to cirrhosis, liver fibrosis, or even liver cancer [7, 8]. The intracellular trafficking of VLDLs is facilitated by specialized VLDL transport vesicles (VTVs) [9, 10], a process that involves specific soluble N-ethylmaleimide-sensitive fusion attachment protein receptor (SNARE) proteins to enable the targeting, docking, and fusion of VTVs with the *cis*-Golgi lumen [9, 11, 12]. However, the specific mechanism whereby the SNARE complex regulates mutual recognition between VTVs and the Golgi apparatus remains to be elucidated.

Sphingosine-1-phosphate (S1P) is a bioactive lipid involved in various physiological processes and human diseases. S1P exerts regulatory effects, including modulation of cell migration, angiogenesis, inflammation, immune cell trafficking, and cholangiocyte proliferation, which are mediated via S1P receptors (S1PRs) [13–16]. S1P is generated by two isoforms of sphingosine kinase (SphK), sphingosine kinase 1 (SphK1) and sphingosine kinase 2 (SphK2) [17]. SphK1 is primarily expressed in the lungs, spleen, thymus, and blood, whereas SphK2 is predominantly expressed in the kidneys and heart, with both being highly expressed in the liver [18]. SphK1 and SphK2 exhibit distinct subcellular localizations, with SphK1 being primarily localized in the cytoplasm and SphK2 in the nucleus, endoplasmic reticulum (ER), and mitochondria [19], indicating that SphK1 and SphK2 may exhibit different, or even contrasting, biological roles owing to the compartment-specific reservoirs of S1P [20]. SphK1 is implicated in the modulation of immune responses and inflammation [21]. In hepatocytes, SphK1 plays a critical role in the initial phase of acute liver failure by promoting ER stress and the mitochondrial permeability transition [22]. Similarly, ER stress is involved in the regulation of hepatic lipid droplets and insulin resistance by SphK2 [23]. However, studies on SphK2 functions, particularly its potential involvement in regulating hepatic lipid metabolism are limited.

Therefore, this study aimed to elucidate the molecular mechanisms through which SphK2 regulates lipid metabolism in the liver. Our analysis indicates that the SphK2-S1P-mTORC2-CMA signaling pathway is a promising therapeutic target for hepatic lipid accumulation.

## Results

### Loss of SphK2 results in TG accumulation in hepatocytes

**Fig. 1:**
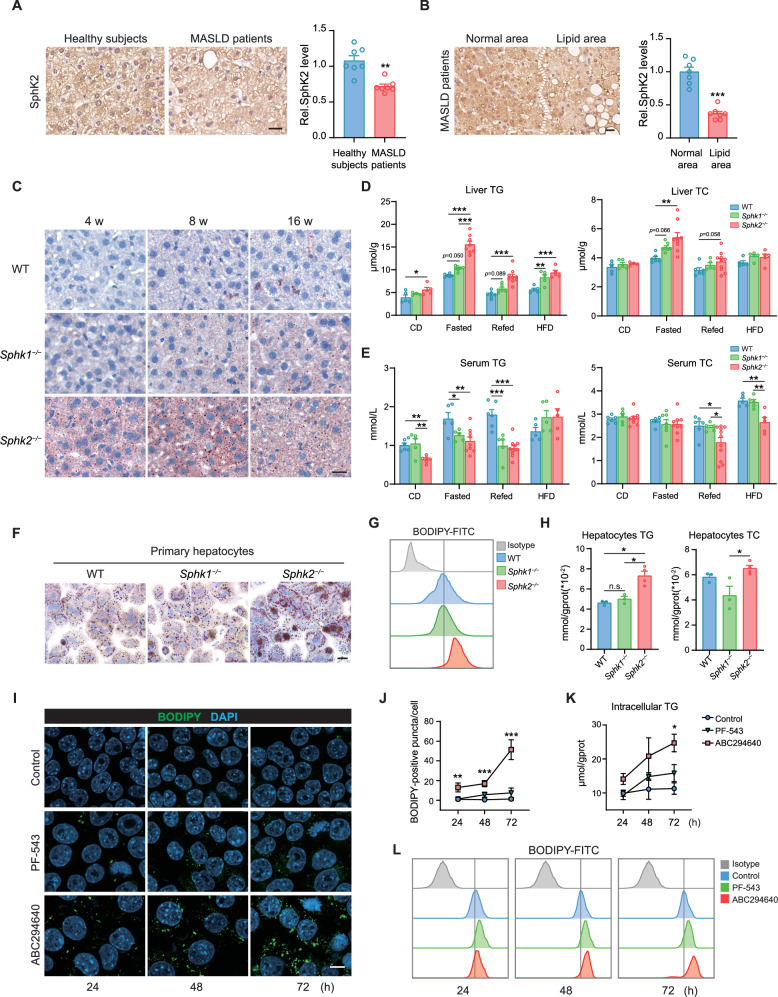
Spontaneous accumulation of TGs in the livers of *Sphk2*^*−/−*^ mice. **A** Representative immunohistochemical images and quantification of SphK2 expression in the livers of patients (*n* = 7) with MASLD and healthy individuals (*n* = 7). Scale bar: 20 μm. Error bars indicate mean ± SEM. **p  <  0.01, unpaired Student’s *t* test. **B** Representative images of immunohistochemical staining and quantification of SphK2 in normal areas and areas adjacent to lipid accumulation (excluding fat vacuoles) in the livers of patients with mild MASLD. Scale bar: 20 μm. Error bars indicate mean ± SEM. ***p  <  0.001, unpaired Student’s *t* test. **C** Oil Red O staining of liver sections from WT, *Sphk1*^*−/−*^, and *Sphk2*^*−/−*^ mice at 4, 8, and 16 weeks of age (*n* = 5‒10 per group). Scale bar: 20 μm. **D** Liver TG and TC levels were measured in 8-week-old WT, *Sphk1*^*−/−*^, and *Sphk2*^*−/−*^ mice subjected to random feeding, fasting, refeeding, or a high-fat diet for 4 weeks (*n* = 4‒10 per group). Error bars indicate mean ± SEM. *p < 0.05, **p < 0.01, and ***p < 0.001, one-way ANOVA. **E** Serum TG and TC levels were quantified in 8-week-old WT, *Sphk1*^*−/−*^, and *Sphk2*^*−/−*^ mice subjected to random CD, fasting, refeeding, or HFD feeding for 4 weeks (*n* = 4‒11 per group). Error bars indicate means ± SEM. *p < 0.05, **p < 0.01, and ***p < 0.001, one-way ANOVA. **F** Oil Red O staining of primary hepatocytes isolated from 8-week-old WT, *Sphk1*^*−/−*^, and *Sphk2*^*−/−*^ mice (*n* = 3‒4 per group); scale bar: 30 μm. **G** Representative histograms of BODIPY 493/503 fluorescence intensity in primary hepatocytes from WT, *Sphk1*^*−/−*^, and *Sphk2*^*−/−*^ mice (*n* = 3‒4 per group). **H** Primary hepatocytes were extracted from WT, *Sphk1*^*−/−*^, and *Sphk2*^*−/−*^ mice (*n* = 3‒4 per group), and TG and TC levels were measured. Error bars indicate mean ± SEM. *p < 0.05, one-way ANOVA. **I**, **J** Representative images of BODIPY 493/503 staining and quantitative analysis of intracellular lipid content in Hepa1-6 cells treated with PF-543 and ABC294640 at concentrations of 10 and 30 μM, respectively, over specified time intervals. Scale bar: 20 μm. Error bars indicate mean ± SEM from three independent experiments. ***p  <  0.001, one-way ANOVA. **K** Intracellular TG content in Hepa1-6 cells was quantified following exposure to 30 μM ABC294640 or 10 μM PF543 for 24, 48, or 72 h. Error bars indicate mean ± SEM from three independent experiments. *p < 0.05, one-way ANOVA. **L** Histogram of BODIPY 493/503-stained Hepa1-6 cells treated with PF543 (10 μM) or ABC294640 (30 μM) for 24, 48, or 72 h. Images represent at least three independent experiments. MASLD metabolic dysfunction-associated steatotic liver disease, TG triglyceride, TC total cholesterol.

To determine the association between SphKs and lipid metabolism in the liver, we examined the expression of SphKs in hepatic tissues of patients with MASLD and healthy individuals. The protein levels of SphK2, but not SphK1, were significantly lower in patients with MASLD than in healthy individuals (Fig. 1A, Supplementary Fig. S1A). Moreover, patients with MASLD exhibited reduced SphK2 levels, particularly in regions proximal to lipid accumulation, with a reduction of > 60% (Fig. 1B, Supplementary Fig. S1B). Consistent with the observations in MASLD patients, high-fat diet (HFD)-fed WT mice exhibited significantly reduced SphK2 levels in the liver, with the lowest level observed at 12 weeks (Supplementary Fig. S1C, D). Accordingly, SphK1 and SphK2 protein levels were significantly reduced in Hepa1-6 cells exposed to palmitic acid (Supplementary Fig. S1E, F). Collectively, these findings suggest a negative correlation between the SphK2 protein levels and lipid homeostasis in liver tissues.

To further investigate the involvement of SphKs in hepatic lipid metabolism, we characterized the hepatic lipid status of *Sphk1*^*−/−*^, *Sphk2*^*−/−*^, and wild-type (WT) mice. Compared with WT mice, *Sphk2*^*−/−*^ mice fed a random chow diet (CD) exhibited significantly increased intrahepatic lipid accumulation, starting at 4 weeks (Fig. 1C). However, the livers of *Sphk1*^*−/−*^ mice showed little lipid accumulation, with a notable increase in the hepatic lipid content at 16 weeks of age (Fig. 1C; Supplementary Fig. S2A). Additionally, *Sphk2*^*−/−*^ mice exhibited significantly higher hepatic TG levels than WT mice under random CD feeding, fasting, refeeding, or four weeks of HFD feeding, whereas *Sphk1*^*−/−*^ mice exhibited notably higher hepatic TG levels than WT mice only during HFD feeding (Fig. 1D). Under normal chow diet conditions, total cholesterol (TC) levels did not differ significantly among these three groups of mice. However, *Sphk2*^*−/−*^ mice exhibited increased TC levels upon fasting (Fig. 1D). Notably, *Sphk2*^*−/−*^ mice exhibited significantly lower peripheral blood TG levels than did WT mice under random CD-feeding, fasting, and refeeding conditions, whereas TG levels in the peripheral blood of *Sphk1*^*−/−*^ mice were significantly lower under fasting and refeeding conditions (Fig. 1E). Moreover, serum TC levels were significantly lower in *Sphk2*^*−/−*^ mice than in *Sphk1*^*−/−*^ and WT mice under refeeding and HFD conditions (Fig. 1E). Overall, these results indicate that SphK2 plays a more significant role in balancing hepatic lipid metabolism than SphK1.

Oil Red O and BODIPY staining revealed that the lipid content in *Sphk2*^*−/−*^ hepatocytes was significantly higher than that in *Sphk1*^*−/−*^ and WT hepatocytes (Fig. 1F, G). Moreover, the levels of TG, but not TC, were elevated in *Sphk2*^*−/−*^ hepatocytes compared with those in WT and *Sphk1*^*−/−*^ hepatocytes (Fig. 1H). Additionally, ABC294640, a specific SphK2 inhibitor, induced significant lipid accumulation in Hepa1-6 cells. However, PF-543, an SphK1-specific inhibitor had no such effect on Hepa1-6 cells (Fig. 1I–L).

Serum levels of aspartate aminotransferase (AST) in 8-week-old *Sphk1*^*−/−*^ and *Sphk2*^*−/−*^ mice fed a random CD did not significantly differ from those in WT mice, although *Sphk2*^*−/−*^ mice exhibited a mild increase in alanine aminotransferase (ALT) levels (Supplementary Fig. S2B). Additionally, the numbers of neutrophils, monocytes, and Kupffer cells infiltrating the livers of *Sphk2*^*−/−*^ mice were not significantly different from those in the livers of WT mice (Supplementary Fig. S2C). Taken together, these findings indicate that TG accumulation in *Sphk2*^*−/−*^ mice does not induce substantial inflammation or cellular damage in the liver.

### Reduced hepatic VLDL-TG secretion in *Sphk2*^*−/−*^ mice

**Fig. 2:**
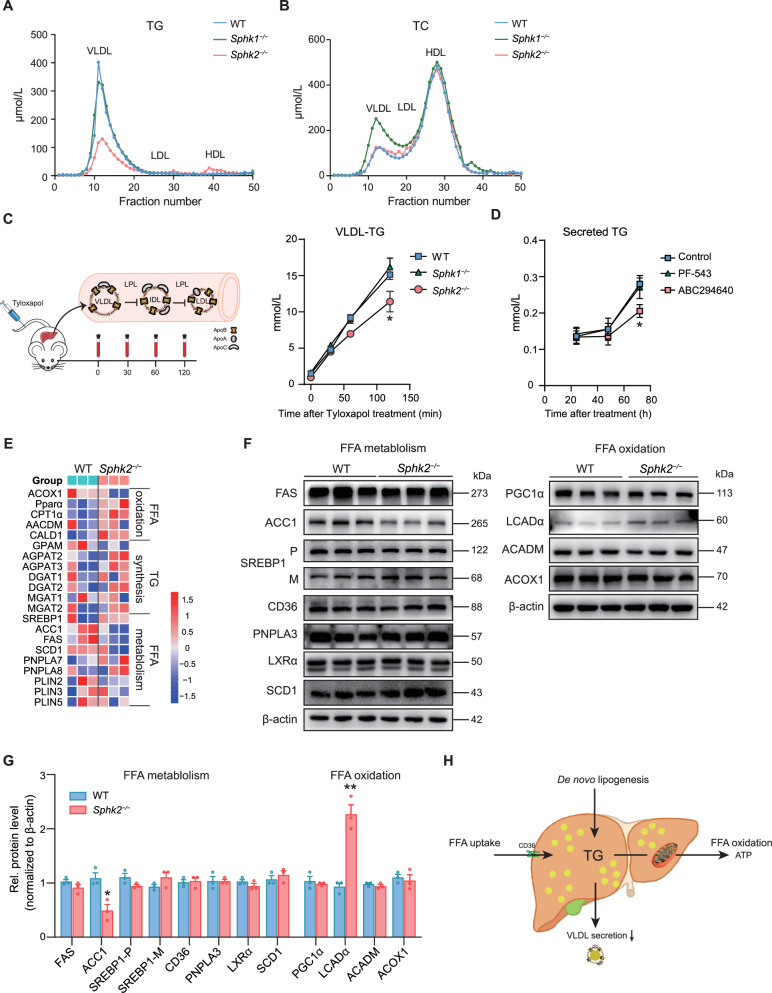
Reduced hepatic VLDL-TG secretion in *Sphk2*^*−/−*^ mice. **A**, **B** Distribution of plasma lipids. The mixed plasma from five mice in each group was fractionated using FPLC, and TG and TC levels were measured in each fraction. **C** Schematic representation of the protocol used to assess VLDL secretion. Blood samples were collected from mice at 0, 30, 60, and 120 min following intravenous injection of tyloxapol (Triton WR-1339) at a dose of 500 mg/kg body weight through the tail vein, after which serum TG levels were measured (*n* = 5 per group). Error bars indicate mean ± SEM. *p < 0.05, one-way ANOVA. **D** The secretion of TG from Hepa1-6 cells into the medium was assessed after treatment with PF-543 or ABC294640 for 24, 48, or 72 h. Error bars indicate mean ± SEM from three independent experiments. *p < 0.05, one-way ANOVA. **E** Heatmap of differentially expressed proteins associated with “FFA oxidation”, “FFA metabolism”, and “TG synthesis” in primary hepatocytes from WT and *Sphk2*^*−/−*^ mice. **F**, **G** Representative western blotting images and quantification of key proteins involved in the “FFA metabolism” and “FFA oxidation” pathways in the livers of WT and *Sphk2*^*−/−*^ mice (*n* = 3 per group). Error bars indicate mean ± SEM. *p  <  0.05 and **p < 0.01, unpaired Student’s *t* test. P, precursor of SREBP1; M, mature SREBP1. **H** Schematic illustration of hepatic TG metabolism and the reduction in VLDL secretion in *Sphk2*^*−/−*^ mice. VLDL very low-density lipoprotein, IDL intermediate-density lipoprotein, LDL low-density lipoprotein, HDL high-density lipoprotein, ACOX1 acyl-CoA oxidase 1, PPARα peroxisome proliferator activated receptor alpha, CPT1α carnitine palmitoyltransferase 1α, ACADM acyl-CoA dehydrogenase medium chain, CALD1 caldesmon 1, GPAM glycerol-3-phosphate acyltransferase, mitochondrial, AGPAT2 1-acylglycerol-3-phosphate o-acyltransferase 2, AGPAT3 1-acylglycerol-3-phosphate o-acyltransferase 3, DGAT1 diacylglycerol o-acyltransferase 1, DGAT2 diacylglycerol o-acyltransferase 2, MGAT1 alpha-1,3-mannosyl-glycoprotein 2-beta-n-acetylglucosaminyltransferase, MGAT2 alpha-1,6-mannosyl-glycoprotein 2-beta-n-acetylglucosaminyltransferase, SREBP1 sterol regulatory element binding transcription factor 1, ACC1 acetyl-CoA carboxylase alpha 1, FAS fatty acid synthase, SCD1 stearoyl-CoA desaturase, PNPLA7 patatin-like phospholipase domain containing 7, PNPLA8 patatin-like phospholipase domain containing 8, PLIN2 perilipin 2, PLIN3 perilipin 3.

Subsequently, we systemically examined the plasma lipid content of *Sphk1*^*−/−*^ and *Sphk2*^*−/−*^ mice. Fast protein liquid chromatography (FPLC) analysis revealed that TG levels in the VLDL fraction, but not in the low-density lipoprotein (LDL) or high-density lipoprotein (HDL) fractions, in *Sphk2*^*−/−*^ mice were significantly lower than those in WT and *Sphk1*^*−/−*^ mice (Fig. 2A). Although no significant differences in TC levels were detected in the serum LDL or HDL fractions among the three groups of mice, TC levels in the VLDL fraction were notably elevated in *Sphk1*^*−/−*^ mice (Fig. 2B). Moreover, we injected tyloxapol into mice to assess the VLDL secretion capacity. A significantly lower hepatic VLDL secretion rate was observed in *Sphk2*^*−/−*^ mice than in WT and *Sphk1*^−/−^ mice (Fig. 2C). Taken together, these results indicate that *Sphk2* deficiency reduces VLDL-TG levels in the peripheral blood. Similarly, ABC294640 treatment significantly reduced VLDL secretion from cultured Hepa1-6 cells (Fig. 2D). These findings suggest that SphK2 plays a pivotal role in VLDL secretion by hepatocytes.

To further determine whether SphK2 is involved in other TG metabolic pathways in the liver, we isolated hepatocytes from *Sphk2*^*−/−*^ and WT mice for proteomic analyses. Principal component analysis (PCA) revealed that the proteomic expression pattern was markedly affected by SphK2 deficiency (Supplementary Fig. S3A). Notably, among the 110 differentially expressed proteins identified, 72 were significantly upregulated and 38 were significantly downregulated (fold change >1.2, *p* < 0.05) in primary hepatocytes from *Sphk2*^*−/−*^ mice (Supplementary Fig. S3B). Furthermore, no differences in the levels of enzymes involved in pathways associated with free fatty acid (FFA) oxidation, FFA metabolism, or TG synthesis were detected between *Sphk2*^*−/−*^ and WT mice (Fig. 2E–G). Reduced levels of ACC1 and increased expression of LCADα were observed in the livers of *Sphk2*^*−/−*^ mice (Fig. 2F, G), suggesting a reduced FFA synthesis and accelerated β-oxidation, possibly owing to overwhelmed TG accumulation. Collectively, SphK2 deficiency led to hepatic lipid accumulation owing to a reduction in hepatic VLDL secretion (Fig. 2H).

### Impaired fusion between VTVs and the Golgi apparatus in the hepatocytes of *Sphk2*^*−/−*^ mice

**Fig. 3:**
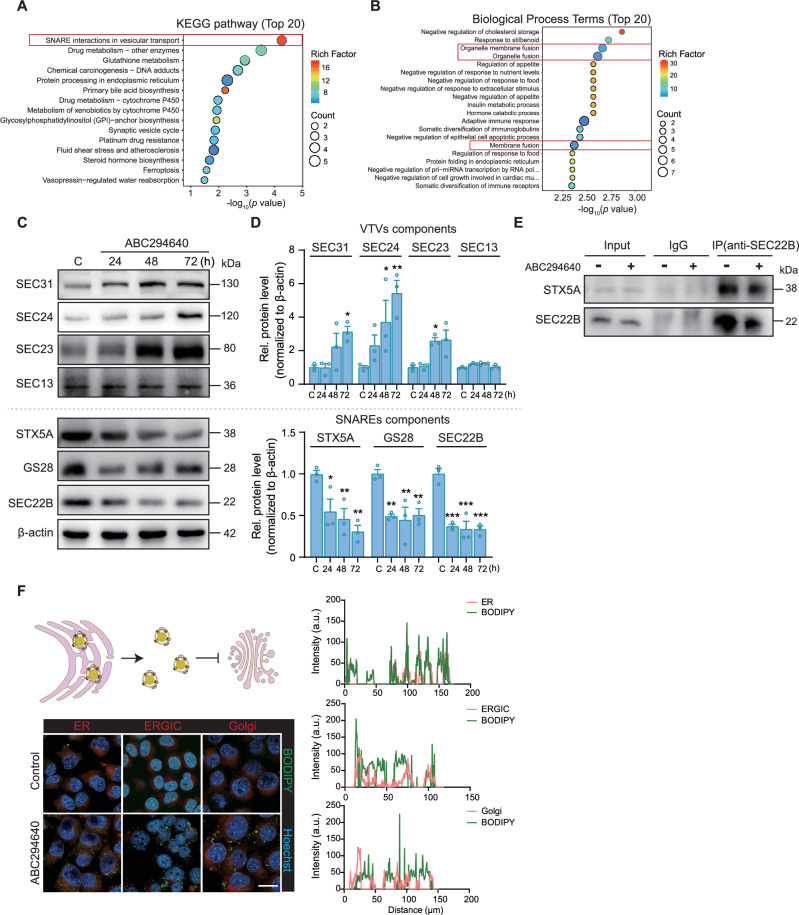
Impaired fusion between VTVs and the Golgi apparatus in the hepatocytes of *Sphk2*^*−/−*^ mice. **A** Kyoto Encyclopedia of Genes and Genomes (KEGG) pathway analysis revealed that the top 20 signaling pathways in which differentially expressed proteins (DEPs) were enriched in primary hepatocytes from WT and *Sphk2*^*−/−*^ mice. **B** Biological processes among the top 20 terms enriched in differentially expressed proteins in primary hepatocytes from the two groups of mice, as determined by GO analysis. **C**, **D** The administration of ABC294640 (30 μM) resulted in substantial upregulation of VTVs (SEC31, SEC24, and SEC23) proteins, accompanied by a notable reduction in the levels of SNARE complex components (SEC22B, STX5A, and GS28) in Hepa1-6 cells. Error bars indicate mean ± SEM from three independent experiments. *p < 0.05, **p < 0.01, and ***p < 0.001, vs. Control, one-way ANOVA. **E** The interaction between STX5A and SEC22B was assessed using coimmunoprecipitation (co-IP). Equivalent protein (800 μg) extracts from Hepa1-6 cells treated with or without ABC294640 were subjected to immunoprecipitation with an anti-SEC22B antibody. The resulting precipitate was subjected to western blotting with antibodies specific for STX5A and SEC22B. Images are representative of three independent experiments. **F** Representative immunofluorescence microscopy images depicting Hoechst 33342 (blue) staining in conjunction with ER-Tracker Red, ERGIC (red), and Golgi-Tracker Red staining. Scale bar: 20 μm; the right panel shows the quantitative analysis of Hepa1-6 cells treated with ABC294640. Images are representative of three independent experiments. SNARE soluble N-ethylmaleimide-sensitive fusion attachment protein receptor, ERGIC endoplasmic reticulum-Golgi intermediate compartment.

Microsomal triglyceride transfer protein (MTP), protein disulfide isomerase (PDI), and secretion-associated Ras-related protein 1 (Sar1) play essential roles in VLDL packaging and efflux from the ER [24, 25]. *Sphk2* deficiency did not notably affect the levels of these proteins (Supplementary Fig. S4A). However, proteomic analysis of the primary hepatocytes of *Sphk2*^*−/−*^ and WT mice revealed that “SNARE interactions in vesicular transport” was the most enriched pathway (Fig. 3A). Additionally, the pathways of “SNARE complex” and “SNARE binding” were enriched in Gene Ontology (GO) terms associated with cellular components and molecular functions, respectively (Supplementary Figs. S4B, C). Among the top 20 differentially enriched biological processes, “organelle membrane fusion”, “organelle fusion”, and “membrane fusion” were the most prominent (Fig. 3B), suggesting that *Sphk2* deficiency affects membrane fusion and recognition processes in hepatocytes.

VLDL transport in hepatocytes relies on the recognition of SNARE complexes, particularly between VTVs and the *cis*-Golgi apparatus [26]. Treatment with ABC294640 led to a progressive increase in the levels of vesicle component proteins SEC31, SEC23, and SEC24 over time, with SEC13 levels showing no changes (Fig. 3C, D). Conversely, the protein levels of the SNARE complex components SEC22B (located on VTVs), STX5A, and GS28 (located on the Golgi membrane) were significantly reduced following 24 h of ABC294640 treatment (Fig. 3C, D). Furthermore, the interaction between SEC22B and STX5A was significantly inhibited (Fig. 3E). Consistent with the above-mentioned findings, the suppression of SphK2 activity by ABC294640 resulted in impaired lipid trafficking between the VTVs and Golgi apparatus, with no observable lipids present in the Golgi apparatus (Fig. 3F).

### SphK2 regulates SNARE stability and accelerates SNARE degradation in a lysosome-dependent manner

**Fig. 4:**
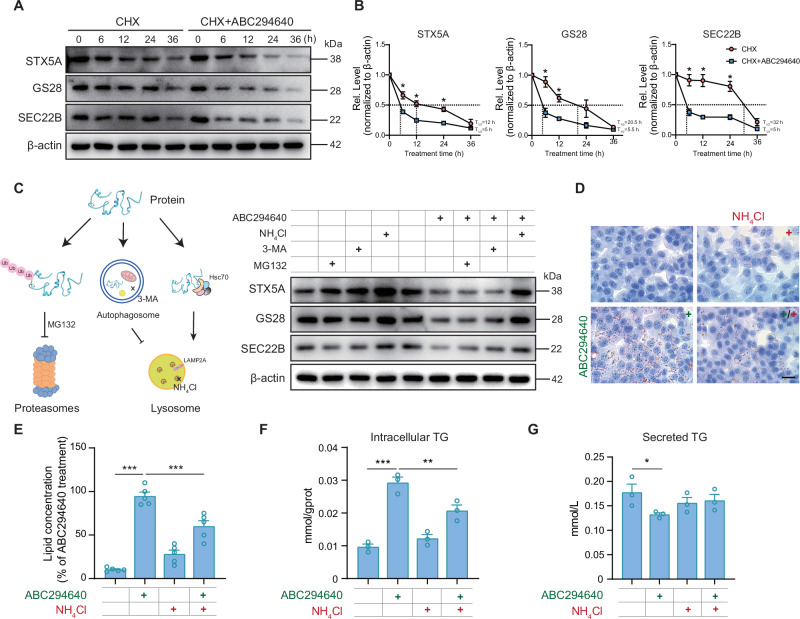
SphK2 regulates SNARE stability and accelerates its degradation via lysosome associated pathways. **A** Hepa1-6 cells were treated with cycloheximide (CHX; 5 mg/mL) in the presence or absence of ABC294640 for various durations. Equal amounts of the cell lysates were blotted using the indicated antibodies. Images are representative of three independent experiments. **B** The STX5A, GS28, and SEC22B signals shown in (**A**) were quantified and plotted. The half-life (T_1/2_) of a protein was defined as the time needed for its concentration to be reduced by 50% relative to the initial level. Error bars indicate mean ± SEM from three independent experiments. *p  <  0.05, unpaired Student’s *t* test. **C** Schematic representation of the protein degradation pathways and principal inhibitors involved (left panel). Representative western blot images showing the levels of STX5A, GS28, and SEC22B in whole-cell lysates from ABC294640-treated Hepa1-6 cells treated with or without the inhibitors NH_4_Cl (8 mM), 3-MA (2.5 mM), and MG132 (10 μM) for 24 h. **D**, **E** Representative images and quantitative analysis of Oil Red O staining in Hepa1-6 cells untreated or treated with ABC294640 and NH_4_Cl. Scale bar: 10 μm. Error bars indicate mean ± SEM from five independent experiments. ***p < 0.001, one-way ANOVA. Images are representative of five independent experiments. **F** Intracellular TG levels were measured in Hepa1-6 cells that were untreated or treated with ABC294640 and NH_4_Cl. **G** The secretion of TG in the medium was also assessed. Error bars indicate mean ± SEM of three independent experiments. *p < 0.05, one-way ANOVA.

Treatment with ABC294640 did not significantly affect the transcript levels of *Sec22b*, *Stx5a*, or *Gs28* (Supplementary Fig. S5). To further explore the mechanism by which SphK2 deficiency affects recognition between VTVs and the Golgi apparatus, we examined the stability of SNARE complex components. The half-lives of STX5A, GS28, and SEC22B were significantly shortened after ABC294640 treatment (Fig. 4A, B). Among the specific inhibitors of major pathways involved in protein degradation (Fig. 4C, Supplementary Fig. S6A–C) [27], NH_4_Cl treatment significantly reversed the reduction in STX5A, GS28, and SEC22B levels induced by ABC294640, whereas MG132 or 3-MA treatment had no ameliorative effect (Fig. 4C). Following treatment with NH_4_Cl, a specific lysosomal function inhibitor, Hepa1-6 cells exhibited minimal lipid accumulation (Fig.4D). Subsequently, the inhibition of SphK2 activity and addition of NH_4_Cl reversed the increase in intracellular lipid and TG accumulation induced by ABC294640 treatment (Fig. 4D–F) and partially improved the reduction in VLDL secretion (Fig. 4G).

These results, together with those of the siRNA-mediated knockdown of *Sphk2* (Supplementary Fig. S7), suggesting that inhibition of SphK2 accelerates the degradation of the SNARE complex and increases intracellular lipids, which are associated with the lysosome pathway.

### LAMP2A or HSC70 knockdown reverses the lipid accumulation caused by *Sphk2* deficiency

**Fig. 5:**
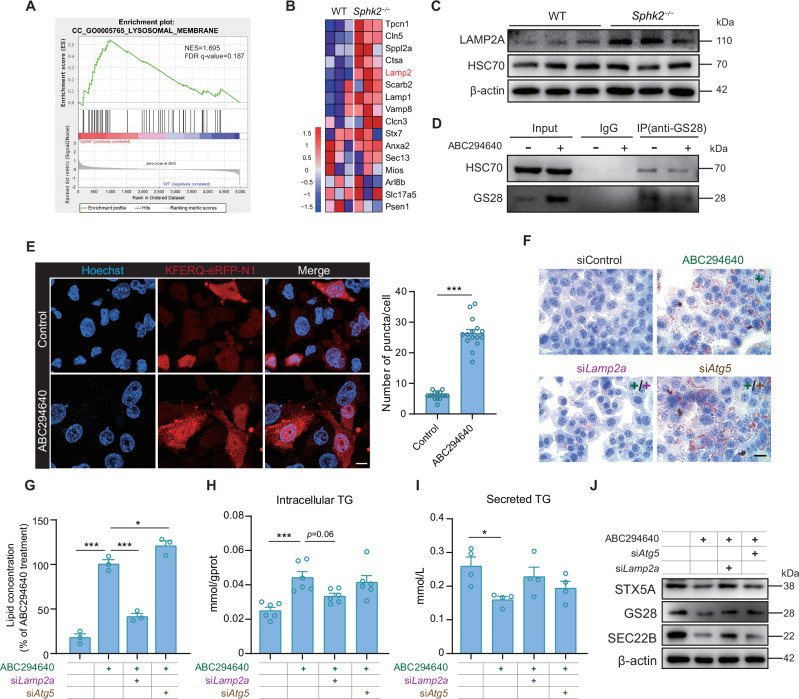
LAMP2A knockdown mitigates TG accumulation induced by *Sphk2* deficiency. **A** Gene set enrichment analysis (GSEA) revealed significant enrichment, particularly in GO terms associated with the lysosomal membrane. **B** Proteins that comprised the leading edge of the enrichment score are shown in the corresponding heatmap. **C** Western blotting was performed to analyze LAMP2A and HSC70 expression in the livers of WT and *Sphk2*^*−/−*^ mice (*n* = 3 per group). **D** Western blot analysis was performed with an anti-GS28 antibody on immunoprecipitated proteins obtained from 800 µg of cell lysates incubated with or without ABC294640 treatment. Images are representative of three independent experiments. **E** Representative immunofluorescence microscopy images and quantification of KFERQ-eRFP-N1 (red) and Hoechst 33342 (blue) staining. The KFERQ-eRFP-N1 protein was expressed in Hepa1-6 cells, and after treatment of Hepa1-6 cells with ABC294640, the number of red fluorescent puncta within the cells significantly increased. Scale bar: 10 μm. Images are representative of three independent experiments. Error bars indicate mean ± SEM from three independent experiments. ***p < 0.001 and unpaired Student’s *t* test. **F** Oil Red O staining of Hepa1-6 cells and its quantification (**G**) after *Atg5* and *Lamp2a* knockdown. Notably, after *Lamp2a* knockdown, the accumulation of lipid droplets in Hepa1-6 cells was significantly reduced compared to that in *Atg5* knockdown cells. Scale bar: 10 μm. Error bars indicate mean ± SEM from three independent experiments. *p < 0.05, **p < 0.01, and ***p < 0.001, one-way ANOVA. Images are representative of three independent experiments. **H** Intracellular and **I** secreted TG levels in Hepa1-6 cells before and after *Atg5* and *Lamp2a* knockdown. Error bars indicate mean ± SEM of four to six independent experiments. *p < 0.05 and ***p < 0.001, one-way ANOVA. **J** Western blotting was performed to analyze STX5A, GS28, and SEC22B levels in Hepa1-6 cells treated or not treated with ABC294640 under *Lamp2a* or *Atg5* knockdown conditions. Images are representative of three independent experiments.

Proteomic analysis revealed a notable increase in pathways associated with lysosomal function in *Sphk2*^*−/−*^ hepatocytes, particularly those related to “lysosomal membranes”, “lysosomes”, and “lysosomal organization” (Fig. 5A, Supplementary Fig. S8A). The levels of proteins involved in macroautophagy, such as Beclin1, p62, and LC3B, did not change significantly under *Sphk2* deficiency (Supplementary Fig. S8B, C). Following treatment with Bafilomycin A1 (BafA1), an inhibitor of lysosomal activities, no significant difference was observed in the autophagic flux of Hepa1-6 cells treated with ABC294640 compared with that of the untreated group (Supplementary Fig. S8D). Notably, elevated LAMP2A levels were observed in the livers of *Sphk2*^*−/−*^ mice (Fig. 5B, C), indicating the activation of CMA. Although *Sphk2*^*−/−*^ mice had a negligible effect on the protein levels of HSC70, a pivotal chaperone protein in the CMA (Fig. 5C), there was an interaction between GS28 and HSC70 (Fig. 5D). After being treated with ABC294640, Hepa1-6 cells exhibited a significant increase in the number of red foci, indicating marked enhancement in CMA activity (Fig. 5E). Furthermore, knockdown of LAMP2A or HSC70 (Supplementary Fig. S9A), critical proteins involved in CMA, significantly reduced lipid and TG accumulation (Fig. 5F–H, Supplementary Fig. S9B–D) and partially restored VLDL secretion (Fig. 5I, Supplementary Fig. S9E) in Hepa1-6 cells treated with ABC294640. Conversely, suppression of the autophagy-associated molecule ATG5 (Supplementary Fig. S9A) did not exhibit a similar restorative effect (Fig. 5F–I, Supplementary Fig. S9B–E). Furthermore, LAMP2A knockdown mitigated the reduction in SNARE protein levels induced by ABC294640 treatment (Fig. 5J), suggesting that the SNARE proteins contain KFERQ-targeting motifs (Supplementary Fig. S9F), including STX5A, GS28, and SEC22B, may serve as potential substrates for chaperone-mediated autophagy (CMA) in the absence of *Sphk2* [28, 29]. Taken together, these findings suggest that lipid accumulation induced by *Sphk2* deficiency can be accomplished with the assistance of CMA.

### mTORC2 activation reverses the hepatocellular lipid accumulation caused by *Sphk2* deficiency

**Fig. 6:**
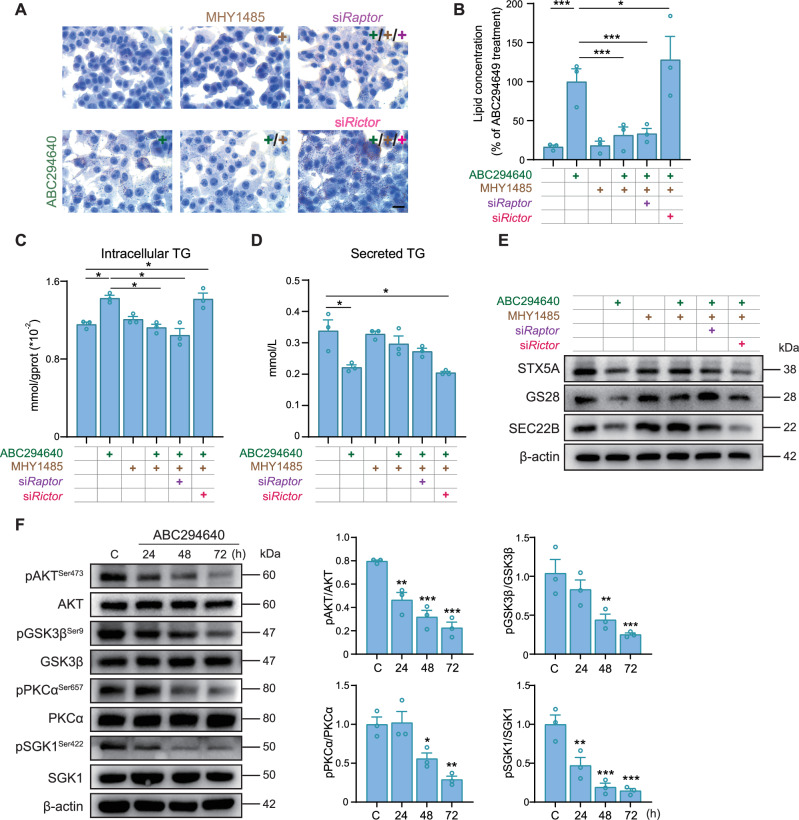
Increased mTORC2 phosphorylation reverses the decrease in VLDL secretion caused by *Sphk2* deficiency. **A** Representative images of Oil Red O staining and **B** quantification of intracellular lipid accumulation in Hepa1-6 cells treated with ABC294640 (30 μM) and MHY1485 in the absence or presence of Raptor (60 pM) and Rictor (60 pM). Scale bar:10 μm. Error bars indicate mean ± SEM from three independent experiments. *p < 0.05 and ***p < 0.001, one-way ANOVA. Images are representative of three independent experiments. **C** TG levels in Hepa1-6 cells and their subsequent secretory capacity (**D**) were assessed following the administration of ABC294640 and MHY1485 in the presence or absence of the regulatory proteins Raptor and Rictor. Error bars indicate mean ± SEM from three independent experiments. *p < 0.05, one-way ANOVA. **E** The expression levels of key proteins, namely STX5A, GS28, and SEC22B, in Hepa1-6 cells were assessed using western blot analysis. Hepa1-6 cells were treated with a combination of ABC294640 (30 μM) and MHY1485 (3 μM) in both the presence and absence of Raptor and Rictor, which are pivotal components of the mTOR complex. Images are representative of three independent experiments. **F** Representative western blots are shown; quantitative analysis was performed to determine the levels of phosphorylated and total AKT, GSK3β, SGK1, and PKCα proteins in Hepa1-6 cells treated with ABC294640 (30 μM) for 24, 48, and 72 h. Error bars indicate mean ± SEM from three independent experiments. *p < 0.05, **p < 0.01, and ***p < 0.001, vs. Control, one-way ANOVA. Images are representative of three independent experiments.

Subsequently, we explored the activation of the CMA pathway in *Sphk2*^*−/−*^ hepatocytes. mTORC2 phosphorylation negatively regulates the activation of the CMA pathway [30]. We hypothesized that the regulatory effect of SphK2 on the CMA pathway in hepatocytes is mediated by mTORC2 activation. MHY1485, a specific activator of mTOR [31], significantly reversed lipid accumulation in ABC294640 treated Hepa1-6 cells (Fig. 6A, B). Furthermore, we selectively silenced the expression of the specific subunits Raptor (in mTORC1) and Rictor (in mTORC2) using siRNA. MHY1485 effectively counteracted the increase in intracellular lipid and TG accumulation caused by ABC294640 even after Raptor silencing. However, when Rictor was silenced, MHY1485 failed to reverse ABC294640 induced intracellular lipid and TG accumulation and markedly increased intracellular lipid levels (Fig. 6A–C). Moreover, silencing Raptor, but not Rictor, in conjunction with MHY1485 treatment, partially reversed the reduction in VLDL secretion caused by ABC294640 treatment (Fig. 6D). mTORC2 facilitates mitochondrial fission and thereby plays a crucial role in the regulation of cellular metabolic homeostasis [32]. To investigate whether SphK2 deficiency affects mitochondrial function, we performed a Seahorse assay and detected no significant differences in the mitochondrial respiratory function (Supplementary Fig. S10A), or glycolytic activity (Supplementary Fig. S10B) between primary WT and *Sphk2*^*−/−*^ hepatocytes. Additionally, JC-1 staining demonstrated that in treated Hepa1-6 cells, JC-1 predominantly formed aggregates, emitting red fluorescence, indicating that the mitochondrial membrane potential was unaffected by ABC294640 treatment (Supplementary Fig. S10C, D). Therefore, SphK2 inhibition did not impair mitochondrial function in hepatocytes. Following Raptor silencing, MHY1485 successfully rescued the ABC294640-induced degradation of the SNARE complex proteins SEC22B, STX5A, and GS28. However, Rictor silencing did not increase the levels of these proteins (Fig. 6E). As expected, ABC294640 treatment suppressed the phosphorylation of protein kinase B (PKB/AKT) at Ser473, glycogen synthase kinase 3β (GSK3β) at Ser9, protein kinase C alpha (PKCα) at Ser657, and serum/glucocorticoid regulated kinase 1 (SGK1) at Ser422 (Fig. 6F), all of which are downstream targets of mTORC2 [33, 34], whereas SphK2 knockdown exhibited a similar inhibitory effect (Supplementary Fig. S11). Together, these findings suggest that the inhibition of SphK2 activity blocks the mTORC2 pathway, thereby facilitating the degradation of SNARE complex proteins via CMA, ultimately leading to TG accumulation in hepatocytes.

### S1P upregulates mTORC2 phosphorylation to maintain intracellular lipid homeostasis

**Fig. 7:**
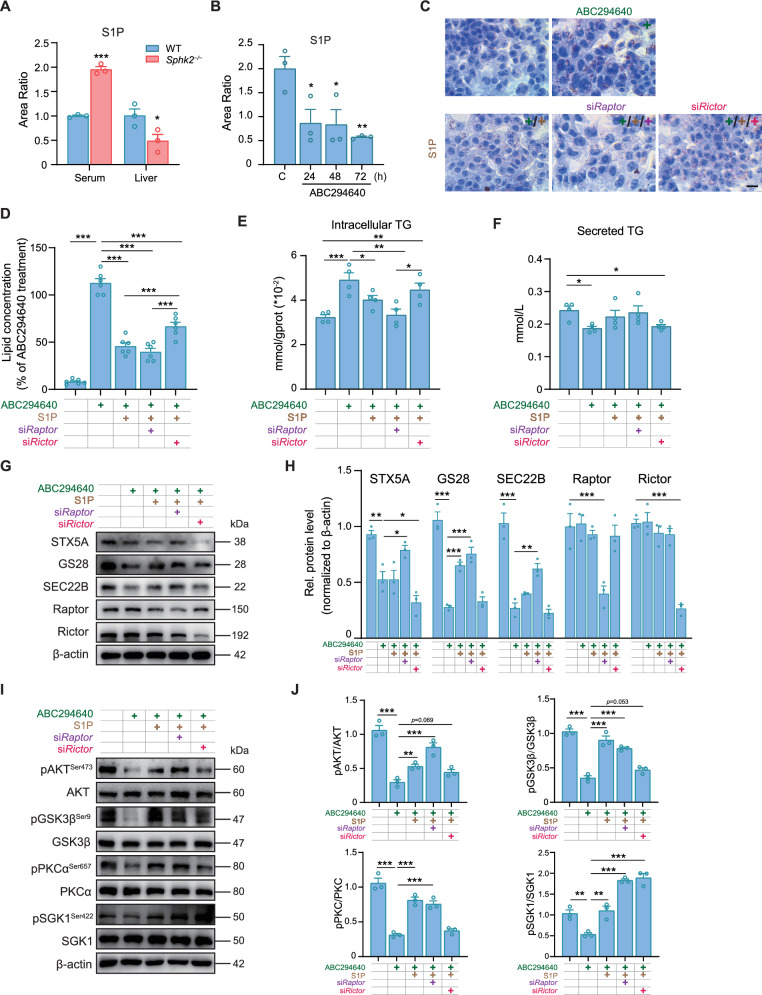
S1P upregulates mTORC2 phosphorylation to maintain intracellular lipid homeostasis. **A** Detection of serum and liver sphingosine 1-phosphate (S1P) concentration area ratios in WT and *Sphk2*^*−/−*^ mice using LC-MS/MS (*n* = 3 per group). Error bars indicate mean ± SEM. *p  <  0.05, ***p  <  0.001, unpaired Student’s *t* test. **B** The area ratio of S1P in Hepa1-6 cells following the administration of ABC294640 for 24, 48, or 72 h. Error bars indicate mean ± SEM from three independent experiments. *p < 0.05 and **p < 0.01, vs. Control, one-way ANOVA. **C** Oil Red O staining and quantification (**D**) in Hepa1-6 cells treated with ABC294640 and S1P in the presence or absence of the mammalian target of the mTORC1 and mTORC2 components Raptor and Rictor. Scale bar: 10 μm. Error bars indicate mean ± SEM from six independent experiments. ***p < 0.001, one-way ANOVA. **E** Intracellular TG levels were quantified in Hepa1-6 cells following treatment with ABC294640 and S1P (2 μM) in the presence or absence of the regulatory proteins Rictor and Raptor. Error bars indicate mean ± SEM from four independent experiments. *p < 0.05, **p < 0.01, and ***p < 0.001, one-way ANOVA. **F** TG secreted by Hepa1-6 cells treated with ABC294640 and S1P in the presence or absence of Rictor and Raptor. Error bars indicate mean ± SEM from four independent experiments. *p < 0.05, one-way ANOVA. **G**, **H** Western blot analysis and quantification of STX5A, GS28, SEC22B, Raptor, and Rictor expression in whole-cell lysates of Hepa1-6 cells treated with ABC294640 or S1P, in the presence or absence of Rictor and Raptor. Error bars indicate mean ± SEM from three independent experiments. *p < 0.05, **p < 0.01, and ***p < 0.001, one-way ANOVA. **I** The expression of AKT, GSK3β, SGK1, PKCα, and their phosphorylated proteins was assessed using western blot analysis. Hepa1-6 cells were treated with the combination of ABC294640 and S1P, in the presence or absence of Raptor and Rictor. The corresponding quantitative histogram is shown in (**J**). Error bars indicate mean ± SEM from three independent experiments. **p < 0.01 and ***p < 0.001, one-way ANOVA.

S1P, catalyzed by SphKs, can activate the mTOR pathway [35]. A notable reduction in S1P content was observed in the livers of *Sphk2*^*−/−*^ mice, whereas S1P levels in the peripheral blood were significantly elevated, possibly attributed to a compensatory mechanism involving increased SphK1 activity (Fig. 7A). ABC294640 also induced a time-dependent decrease in the intracellular S1P content in Hepa1-6 cells in vitro (Fig. 7B). Supplementation with exogenous S1P significantly reversed ABC294640-induced lipid accumulation, whereas activation of S1PR1 or S1PR2 with BAF312 or CYM5520, respectively, failed to ameliorate this effect (Supplementary Fig. S12A, B). Furthermore, Raptor silencing exacerbated the reduction in intracellular lipid accumulation induced by ABC294640 following S1P treatment (Fig. 7C, D). S1P administration promoted TG secretion, thereby substantially reducing intracellular TG accumulation caused by ABC294640. Silencing Rictor but not Raptor significantly blocked these effects (Fig. 7E, F). Moreover, S1P supplementation slightly increased SEC22B and GS28 levels. Although Raptor silencing improved SEC22B and STX5A protein levels, S1P supplementation and Rictor silencing failed to restore normal levels of these proteins in hepatocytes (Fig. 7G, H). Conversely, exogenous S1P significantly increased the phosphorylation of AKT, GSK3β, PKCα, and SGK1 in the mTORC2 pathway (Fig. 7I, J). Taken together, these results indicate that the inhibition of SphK2 activity results in reduced hepatic S1P production, subsequently downregulating the mTORC2 pathway. This downregulation accelerated the degradation of the SNARE complex components, SEC22B, GS28, and STX5A, thereby hindering the fusion of VTVs with the Golgi apparatus and ultimately reducing VLDL secretion by hepatocytes (Graphical Abstract).

## Discussion

The involvement of SphKs in preserving hepatic homeostatic equilibrium has been well documented. The current study revealed a significant reduction in the hepatic VLDL secretion capacity in *Sphk2*^*−/−*^ mice, accompanied by accelerated CMA degradation involving the SNARE complex components SEC22B, STX5A, and GS28. Consequently, these alterations affect mutual recognition between VTVs and the Golgi apparatus as well as the subsequent extracellular excretion of VLDL. Notably, the upregulation of mTORC2 phosphorylation restored the ability of cells to secrete VLDL, thereby markedly improving lipid accumulation in hepatocytes following SphK2 inhibition. S1P, the catalytic product of SphK2, upregulated mTORC2 phosphorylation, impeded the degradation of the SNARE complex via CMA, and promoted the secretion of VLDL from hepatocytes. In summary, our study revealed that SphK2 is a crucial member of the VLDL metabolic regulatory network.

Sphingosine is phosphorylated by SphK1 and SphK2, resulting in the production of S1P, which exerts diverse biological effects. Compared with WT mice, *Sphk2*^*−/−*^ mice showed a significant decrease in the hepatic S1P content. Conversely, S1P levels were substantially higher in the peripheral blood of *Sphk2*^*−/−*^ mice than in that of WT mice. This discrepancy may be attributed to a compensatory increase in SphK1 activity caused by SphK2 deficiency. This is because SphK1 and SphK2 exhibit distinct subcellular localizations and functions in hepatic cells [20]. S1P, catalyzed by SphK1, is secreted into the extracellular space, especially into the blood, where it interacts with S1PRs to mediate various biological functions [36]. SphK2 is predominantly localized in the nucleus, ER, and mitochondria. Within these subcellular compartments, the enzymatic activity of SphK2 facilitates the mediation of diverse biological functions through the production of S1P [17]. Supplementation with exogenous S1P rapidly activated mTORC2 phosphorylation, leading to a reduction in cellular lipid accumulation induced by SphK2 deficiency. However, the S1PR agonists BAF312 and CYM5520 failed to improve the lipid accumulation induced by ABC294640. These findings indicate that S1P activates the mTORC2 pathway and reduces hepatic lipid accumulation through a mechanism independent of S1PRs.

VLDL secretion provides a major pathway for the transport and clearance of intracellular TG from hepatocytes into peripheral circulation [37]. In eukaryotic cells, sorting, packaging, and targeted delivery of numerous substances, including VLDLs, relies on vesicle transport [9, 38]. Proteomic analysis of primary mouse hepatocytes revealed that SphK2 deficiency hindered subcellular organelle and membrane fusion, indicating the potential involvement of SphK2 in regulating intracellular membrane fusion. The formation of a distinctive SNARE complex, involving SEC22B on the surface of VTVs and t-SNAREs (GS28, STX5A, and rBet1) on the *cis*-Golgi apparatus of hepatocytes, facilitates the entry of VLDL into the Golgi body for subsequent modifications [26]. Notably, the absence of SphK2 expedited the degradation of SEC22B, STX5A, and GS28, which are integral components of the SNARE complex, in a CMA-dependent manner.

In addition to its association with neurodegenerative diseases and cancer, CMA actively participates in regulating lipid metabolism [39]. CMA facilitates the degradation of lipogenic enzymes, lipid carriers, and lipid droplet coat proteins involved in lipid metabolism [29]. The conventional viewpoint suggests that lipophagy, a biological process classified as a subtype of autophagy, enables lipid internalization by intracellular autophagic vacuoles, thereby accelerating lipid degradation [40]. Notably, a significant increase in lipid accumulation was observed in the hepatocytes of *Sphk2*^*−/−*^ mice, although no changes in lipophagy were detected. In contrast, the hepatocytes of *Sphk2*^*−/−*^ mice exhibited significantly higher levels of LAMP2A than WT mice, and CMA was significantly activated in Hepa1-6 cells treated with ABC294640. Furthermore, inhibition of the CMA pathway substantially reduced lipid accumulation in *Sphk2*^*−/−*^ hepatocytes and reversed alterations in the protein levels of the SNARE complex constituents SEC22B, STX5A, and GS28. These findings suggest that the components of the SNARE complex serve as substrates for CMA under certain conditions. These results did not confirm whether CMA activated by SphK2 deficiency could regulate the degradation of other components of the SNARE complex. Therefore, further investigation into the involvement of SphK2 in regulating membrane fusion, particularly its effect on CMA activity, is warranted.

mTOR, an atypical serine/threonine kinase, is present in two distinct mammalian cell complexes: mTORC1 and mTORC2 [41]. Previous studies have indicated a close association between the mTOR pathway and autophagy. mTORC1 plays a pivotal role in the regulation of various aspects of macroautophagy, including autophagosome elongation, maturation, and termination [42]. Conversely, mTORC2 negatively influences the formation of the CMA translocation complex by modulating GFAP phosphorylation, which is dependent on AKT [30]. However, previous studies have indicated that mTORC2 influences macroautophagy through PKC [43], suggesting that alterations in mTORC2 substrates facilitate the coordination of macroautophagy and CMA activity. Following the inhibition of SphK2 activity, the mTORC2/AKT pathway was downregulated. By specifically increasing the level of mTORC2 phosphorylation without affecting mTORC1, the enhanced CMA activity in cells due to the inhibition of SphK2 activity can be ameliorated, thereby restoring the protein levels of the SNARE complex components and ultimately promoting VLDL secretion. The substrate S1P could function as a regulator of the mTORC2/AKT/CMA pathway; however, phosphorylated mTORC2 elicited a substantially greater improvement than S1P supplementation in a model of intracellular lipid accumulation caused by *Sphk2* deficiency. Further studies are required to determine the involvement of other kinases in this process.

Collectively, our results highlight an important role for SphK2 in maintaining intracellular lipid homeostasis, particularly in preserving intracellular membrane fusion. *Sphk2* deficiency inhibits mTORC2 phosphorylation, thereby facilitating the degradation of SEC22B, STX5A, and GS28, which are integral components of the SNARE complex, through CMA. This process hinders the translocation of VTVs within cells. Furthermore, our findings reveal a unique biological function of SphK2 and its potential therapeutic value in activating CMA and regulating mTORC2 phosphorylation.

## Methods and materials

### Human participants

Liver samples were obtained from healthy individuals and 14 patients with biopsy-confirmed MASLD at the First Affiliated Hospital of the Anhui Medical University. The research was conducted in accordance with both the Declarations of Helsinki and Istanbul. All procedures pertaining to the use of human samples were approved by the Ethics Committee of Anhui Medical University (PJ-YX2024-042). The clinical data of all the participants are summarized in Supplementary Table 1.

### Animals and diets

Male mice lacking SphK1 (*Sphk1*^*−/−*^) or SphK2 (*Sphk2*^*−/−*^) on a C57BL/6J background were purchased from the Jackson Laboratory (Bar Harbor, ME, USA). Male C57BL/6J mice were obtained from Shanghai Silaike Experimental Animal Co., Ltd. (Shanghai, China). The mice were housed in a barrier facility under specific pathogen-free conditions and a 12-h light/dark cycle at 25 °C. The mice were randomly assigned to the following experiments. Eight-week-old male C57BL/6J mice were fed a high-fat diet (HFD) (60% kcal% fat; catalog no. D12492; Research Diet, New Brunswick, NJ, USA) or chow diet for 4, 8, or 12 weeks. Unless otherwise specified, mice were provided *ad libitum* access to regular chow and water.

Additionally, 4-week-old *Sphk1*^*−/−*^, *Sphk2*^*−/−*^, and WT mice were fed an HFD for 4 weeks to investigate the mechanisms underlying lipid metabolism. *Sphk1*^*−/−*^, *Sphk2*^*−/−*^, and WT mice were subjected to fasting and refeeding for 24 h. Mice in the refeeding group were allowed to refeed for 4 h following the initial 24-h fasting period [44]. Subsequently, the mice were euthanized with tribromoethanol, and peripheral blood and liver tissues were collected. Liver tissue was perfused with phosphate-buffered saline (PBS) to eliminate circulating blood. All experimental procedures were approved by the Laboratory Animal Care and Use Committee of Anhui Medical University (LLSC20200327).

### Cell culture

Two-step perfusion with pronase–collagenase (Sigma-Aldrich, St. Louis, MO, USA) was used to isolate primary hepatocytes from the livers of WT, *Sphk1*^*−/−*^, and *Sphk2*^*−/−*^ mice fed a chow diet (CD). Hepatocytes were subsequently cultured as previously described [45]. Hepa1-6 cells (ATCC, Manassas, VA, USA) were cultured in Dulbecco’s modified Eagle’s medium (DMEM; Thermo Fisher Scientific, Carlsbad, CA, USA) supplemented with 10% (v/v) fetal bovine serum (Thermo Fisher Scientific). The cells underwent STR profiling for authentication and were tested negative for mycoplasma contamination. To block SphK1 and SphK2 activities, Hepa1-6 cells were treated with PF-543 (10 μM) [46] or ABC294640 (30 μM) [47] for 0, 24, 48, or 72 h. Hepa1-6 cells were treated with 0.6 mM palmitic acid for 24 h to induce intracellular lipid accumulation. Proteasomal/lysosomal inhibitor treatment was performed by treating cells with MG132 (MedChemExpress, MCE, New Jersey, USA, 10 μM), 3-MA (MCE, 2.5 mM), or NH_4_Cl (MCE, 8 mM) for 24 h. All concentrations were the final concentrations in the culture medium.

### Protein isolation and western blotting

Protein extracts were obtained from cell and liver tissue samples using radioimmunoprecipitation lysis buffer supplemented with protease and phosphatase inhibitors (Roche, Basel, Switzerland) and quantified using a bicinchoninic acid assay (BCA; NCM Biotech, Suzhou, China). Proteins were separated using 10% sodium dodecyl sulfate‒polyacrylamide gel electrophoresis and transferred onto polyvinylidene fluoride membranes (Merck Millipore, Burlington, VT, USA), which were blocked with 5% nonfat milk. Proteins were detected using primary antibodies (Supplementary Table 2) and horseradish peroxidase-conjugated secondary antibodies, followed by visualization using an enhanced chemiluminescence (ECL) western blotting detection system (Vilber, Marne-la-Vallée Cedex 3, France). Original western blots for the results are provided in Supplementary materials.

### Secretion of VLDL-TAG in mice and Hepa1-6 cells

Eight-week-old male WT, *Sphk1*^*−/−*^, and *Sphk2*^*−/−*^ mice were fasted for 8 h and injected with tyloxapol (Triton WR-1339, 500 mg/kg dissolved in PBS) through the tail vein, as previously described [48]. Blood was collected from the retro-orbital plexus at the indicated time points, and serum TG levels were measured. Untreated or ABC294640-treated Hepa1-6 cells were incubated for 6 h in DMEM supplemented with 0.5% fatty acid-free BSA, washed with PBS, and incubated for an additional 16 h in serum-free and phenol-red-free DMEM, as previously described [49]. The cells and media were then collected and assayed for TG using an enzymatic TG assay kit (Nanjing Jiancheng Bioengineering Institute, Nanjing, China).

### RNA isolation and quantitative PCR analysis

Total RNA was extracted from Hepa1-6 cells using TRIzol reagent (Thermo Fisher Scientific). Subsequently, cDNA was synthesized via the reverse transcription of 1–2 μg of RNA using a reverse transcription kit (Merck KGaA, Darmstadt, Germany). Real-time PCR was performed using SYBR Green on a Roche LightCycler 96 qPCR System (Roche). The sequences of all the primers used in this study are listed in Supplementary Table 3.

### Statistical analysis

Statistical analysis was conducted using an unpaired Student’s *t* test or one-way analysis of variance, followed by Tukey’s post hoc test for comparisons between two or three groups. Data were analyzed using GraphPad Prism version 8 (GraphPad Software Inc., San Diego, CA, USA). Statistical significance was set at *p* < 0.05. The investigator was blinded to group allocation during the experiment and data collection.

## Supplementary information

Supplementary information for Sphingosine kinase 2 deficiency impairs VLDL secretion by inhibiting mTORC2 phosphorylation and activating chaperone-mediated autophagy

Original Data Files

## Data Availability

All data needed to evaluate the conclusions in the paper are present in the paper and/or the Supplementary Materials. Additional data related to this paper may be requested from the authors. Hepatic proteomic data are available via ProteomeXchange with identifier PXD059594.
