# Supplementary material for: Sphingosine kinase 2 deficiency impairs VLDL secretion by inhibiting mTORC2 phosphorylation and activating chaperone-mediated autophagy
Source: Cell Death Differ. 2025 Apr 8;32(10):1886–99. doi: 10.1038/s41418-025-01507-6 (PMC12500862; doi:10.1038/s41418-025-01507-6)
Supplement: Supplementary file 1 — Supplementary information for Sphingosine kinase 2 deficiency impairs VLDL secretion by inhibiting mTORC2 phosphorylation and activating chaperone-mediated autophagy [file 41418_2025_1507_MOESM1_ESM.pdf]

**SUPPLEMENTARY INFORMATION**

# Sphingosine kinase 2 deficiency impairs VLDL secretion by inhibiting mTORC2 phosphorylation and activating chaperone-mediated autophagy

Shuangshuang Zhang[1†], Gaoxiang Li[1†], Lianping He[2†], Fei Wang[1], Mengru Gao[3, 4], Tianliang Dai[1], Yushuang Su[1], Luyan Li[1], Ying Cao[1], Minghua Zheng[5, 6], Liang Chen[1], Jun Cao[1], and Hong Zhou[1*]

[1]School of Life Sciences, Anhui Medical University, Hefei, Anhui 230032, China

[2]Department of Immunology, Nanjing Medical University, Nanjing 211166, China

[3]Clinical Pathology Center, the First Affiliated Hospital of Anhui Medical University, Hefei, Anhui 230012, China

[4]Anhui Public Health Clinical Center, Hefei, Anhui 230012, China

[5]NAFLD Research Center, Department of Hepatology, the First Affiliated Hospital of Wenzhou Medical University, Wenzhou 325000, China

[6]Key Laboratory of Diagnosis and Treatment for The Development of Chronic Liver Disease in Zhejiang Province, Wenzhou, 325000, China

† These authors contributed equally to this work.

## Supplementary Methods and Materials

### Flow cytometry and antibodies

Cell suspensions were incubated with 100 μL of 0.1 μg/mL boron dipyrromethene (BODIPY) 493/503 staining solution (Sigma-Aldrich) to analyze the intracellular lipid content in primary hepatocytes and Hepa1-6 cells. Leukocytes in the liver were collected as described previously [1] and resuspended in cold PBS supplemented with 3% rat serum, followed by incubation with PE-conjugated anti-CD45, FITC-conjugated anti-CD11b, APC-conjugated anti-F4/80, PC7-conjugated anti-Ly6G, Pacific blue-conjugated anti-Ly6C antibodies (BioLegend, California, USA), and diamidino-2-phenylindole (DAPI; Abcam) to analyze the frequency of hepatic immune cells. Flow cytometric analysis was performed using the Beckman Cytoflex S system (Beckman, Brea, USA), and the data were processed using CytoExpert V2.4 (Beckman) and FlowJo V10 (Stanford University, USA).

### Plasma lipid analysis

Pooled sera (500 μL) from five mice in each group were fractionated by FPLC using a Superose 6 PC 3.2/30 column (GE Healthcare Europe GmbH, Munich, Germany) [2]. The serum was fractionated at a constant flow rate of 50 μL/min with PBS, and the TC and TG contents in each fraction were measured according to the manufacturer's instructions (Nanjing Jiancheng Bioengineering Institute).

### Transient transfection

Small interfering RNAs (siRNAs) including LAMP2 siRNA (cat. no. sc-35791) and Atg5 siRNA (cat. no. sc-341446) and HSC70 siRNA (cat. no. sc-44303) and Rictor siRNA (cat. no. sc-61479) and Raptor siRNA (cat. no. sc-108002), or SphK2 siRNA (cat. no. sc-39226) or control siRNA (cat. no. sc-36869, and sc-37007) were purchased from Santa Cruz

Biotechnology, Inc. Briefly, 24 h prior to transfection, the cells were seeded into a 6-well plate to reach a confluence of 30–40%. Subsequently, 6 μL of 10 μM siRNA was mixed with 6 μL of siRNA Transfection Reagent (Santa Cruz Biotechnology, Inc.) in 200 μL of siRNA Transfection Medium, followed by an incubation for 40 min at 25°C. The mixtures were added to the cells for 6–8 h and then replaced with fresh DMEM supplemented with 10% FBS, and the cells were cultured for a certain period for subsequent experiments.

**Coimmunoprecipitation (co-IP)**

Hepa1-6 cells treated with or without ABC294640 for 24 h were harvested and lysed in IP lysis buffer. The protein concentrations in the supernatants were quantified using a BCA assay. For immunoprecipitation, 800 μg of protein was incubated overnight with agarose-conjugated anti-SEC22B (Abcam), anti-GS28 (Santa Cruz Biotechnology, Inc.), or IgG (Proteintech, Wuhan, China) at 4°C. Subsequently, the immunoprecipitated beads were eluted to obtain the immune complex and subjected to western blot analysis.

**Hepatic proteomic analysis**

Proteomic analysis was performed on primary hepatocytes from wild-type (WT) and *Sphk2*$^{-/-}$ mice. Samples were lysed and quantified using a commercial BCA kit (Merck KGaA). Trypsin digestion was performed, and the resulting peptides were labeled with a tandem mass tag (TMT) reagent according to the manufacturer's instructions (Thermo Fisher Scientific). The labeled peptides were separated using reverse-phase high-performance liquid chromatography (Pierce™ high-pH reversed-phase peptide fractionation) and resuspended in 1% formic acid. All samples were analyzed using a liquid chromatography-mass spectrometry (LC-MS) data acquisition strategy on a Q-Exactive HF-X mass spectrometer (Thermo Fisher Scientific) equipped with an Easy nLC 1200 chromatography system (Thermo Fisher Scientific) for

sample processing and peptide separation. Raw data were imported into the Sequest HT search engine using the Proteome Discoverer software (Thermo Fisher Scientific, version 2.4) for database retrieval. The database used for the search was UniProt-*Mus musculus* (Mouse) [10090]-88037-20220325, which was obtained from the protein database at https://www.uniprot.org/taxonomy/10090. Finally, the quantified proteins were subjected to pathway analysis.

**Histological analysis**

Briefly, mice were perfused with 10 mL of ice-cold PBS, and the excised liver tissues were fixed with 10% formalin, embedded in paraffin, and sectioned (Leica, Wetzlar, Germany) to a thickness of 5 μm. H&E staining (Sigma-Aldrich) was performed on tissue sections. For the analysis of SphK levels in the liver tissue, the sections were incubated with a monoclonal anti-SphK antibody and subsequently treated with goat anti-mouse anti-rabbit IgG/IgM H & L (HRP polymer, Abcam). The sections were then treated with a diaminobenzidine solution (DAB-34002; Thermo Fisher Scientific). Frozen sections (10 μm) were used for Oil Red O staining. Additionally, Oil Red O staining of the cells was performed according to the instructions provided with the staining kit (Solarbio, Beijing, China). SphK and cellular lipid levels were quantified using Image-Pro Plus software (Media Cybernetics Inc., Bethesda, MD, USA).

**Immunofluorescence microscopy**

Cells were cultured in glass-bottom culture dishes (Corning, New York, USA) and fixed with 4% paraformaldehyde for 15 min. Subsequently, the cells were permeabilized using 3% Triton X-100, blocked with 5% fetal goat serum, and incubated overnight with primary antibodies (Supplementary Table S2). Subsequently, the cells were incubated with the following fluorescently labeled secondary antibodies: Cy3-conjugated goat anti-rabbit IgG (H-L), Alexa

Fluor 488-conjugated goat anti-rabbit IgG (H-L), and Alexa Fluor 488-conjugated goat anti-mouse IgG (H-L) (Abcam). Finally, the cell nuclei were stained with DAPI (Abcam) or Hoechst 33342 (Sigma-Aldrich), followed by image acquisition using an LSM800 confocal microscope (Carl Zeiss, Oberkochen, Germany) and processing using LSM Zen 3.3 software (Carl Zeiss AG).

**Protein stability assay**

The stability of STX5A, GS28, and SEC22B in Hepa1-6 cells was determined using a cycloheximide (CHX)-based protein chase experiment, as described previously [3]. Total cell lysates were analyzed using western blotting. For quantitative analysis, western blot bands were quantified using ImageJ software (Bio-Rad, Hercules, CA, USA).

**CMA assay**

To confirm the activation of CMA by *Sphk2* deficiency, we used a specific fluorescent CMA reporter as previously described[4]. The recombinant lentiviral vector HBLV-KFERQ-eRFP-N1 was constructed with a backbone encoding an enhanced red fluorescent protein (eRFP) fused to a KFERQ-like motif. Lentiviral packaging was performed by Hanbio (China), yielding a viral titer of $1.5\times10^8$ TU/mL. Hepa1-6 cells were cultured to 50%-70% confluence and infected with HBLV-KFERQ-eRFP-N1 lentivirus (MOI=30), supplemented with 10 μg/mL polybrene. After 12 h of incubation, the medium was replaced with a fresh culture medium. After 72 h of infection, eRFP expression was assessed by confocal microscopy. If the infection efficiency was insufficient, the cells were cultured in medium containing puromycin (2 μg/mL) until the proportion of red fluorescent cells exceeded 80%, at which point they were used for subsequent experiments. After 24 h of treatment with ABC294640, the cell nuclei were labeled with Hoechst, and the eRFP signal in the cells was analyzed using an LSM800 confocal

microscope (Carl Zeiss) and processed using the LSM Zen 3.3 software (Carl Zeiss AG).

**Mitochondrial stress assay**

Cellular oxygen consumption rate (OCR) was measured using a Seahorse XFe24 Analyzer (Agilent Technologies). Briefly, WT and *Sphk2*$^{-/-}$ primary hepatocytes were seeded in XFe24 cell culture microplates (#100777-004, Agilent Technologies) in DMEM supplemented with 10% FBS. The cells were cultured at 37°C in a humidified incubator with 5% $CO_2$ until fully adhered. Prior to measurement, the medium was replaced with 500 μL of pre-warmed, serum-free, unbuffered Seahorse XF base medium (#103334-100, Agilent Technologies), pH 7.4, which had been pre-heated to 37°C and supplemented with reagents for mitochondrial oxidative metabolism analysis. The cell culture microplates were incubated in a non-$CO_2$ incubator at 37°C for 1 h. The cartridge containing the oxygen and pH-sensitive probes was pre-incubated overnight in a non-$CO_2$ incubator at 37°C with a calibration solution (#100840-000, Agilent Technologies). OCR and extracellular acidification rate (ECAR) were measured before and after the injection of the following compounds: OCR measurements (#103015-100, Agilent Technologies): 1 mM oligomycin, 0.5 mM FCCP [carbonyl cyanide-4-(trifluoromethoxy)phenylhydrazone], and 0.5 mM antimycin A. ECAR measurements (#103020-100, Agilent Technologies): 10 mM glucose, 1 μM oligomycin, and 50 mM 2-deoxyglucose (2-DG).

**JC-1 staining**

Hepa1-6 cells treated or untreated with ABC294640 for 24 h were collected. The positive control group was pretreated with CCCP (50 μM) for 5 min. The cells from each group were stained with JC-1 dye (2 μM) and incubated at 37°C for 20 minutes. After staining, the cells were washed several times with cold PBS to remove the excess dye. Subsequently, the cells

were analyzed by flow cytometry to assess the red and green fluorescence intensities of JC-1 staining. Red fluorescence (JC-1 aggregates) indicates high mitochondrial membrane potential, whereas green fluorescence (JC-1 monomers) indicates low mitochondrial membrane potential.

**LC-MS/MS analysis of S1P**

Serum and liver tissue samples were collected from 8-week-old male WT and *Sphk2*$^{-/-}$ mice, and Hepa1-6 cells treated with ABC294640 were analyzed. Plasma (50 μL) was mixed with an equal volume of methanol and acetonitrile solution (200 μL), and the liver tissue and Hepa1-6 cell homogenate were mixed with five volumes of 50% methanol. Analytes were separated on a reverse-phase C18 column (Acquity UPLC BEH C18 Column, 130 Å, 1.7 μm, 2.1 mm × 50 mm) using a gradient of buffers A (water/formic acid 99.9/0.1 (v/v)) and B (acetonitrile) with a flow rate of 0.6 mL/min. The column was maintained at 40°C, and the total analysis time was 3.2 min. The analytes were ionized by electrospray ionization operating in the positive ionization mode, and the scanning mode was multiple reaction monitoring. Data were processed using the Analyst 1.7.2 software system.

**Reference:**

1. Shi W, Wang Y, Zhang C, Jin H, Zeng Z, Wei L, *et al.* Isolation and purification of immune cells from the liver. *Int Immunopharmacol* 2020, 85**:** 106632.

2. Wiesner P, Leidl K, Boettcher A, Schmitz G, Liebisch G. Lipid profiling of FPLC-separated lipoprotein fractions by electrospray ionization tandem mass spectrometry. *J Lipid Res* 2009, 50(3)**:** 574-585.

3. Chan SC, Lin SC, Li P. Regulation of Cidea protein stability by the ubiquitin-mediated proteasomal degradation pathway. *Biochem J* 2007, 408(2)**:** 259-266.

4. Koga H, Martinez-Vicente M, Macian F, Verkhusha VV, Cuervo AM. A photoconvertible fluorescent reporter to track chaperone-mediated autophagy. *Nat Commun* 2011, 2**:** 386.

## Supplemental Figures legends

**Figure S1. Hepatic lipid accumulation is accompanied by decreased SphK protein levels**

**A** Representative images of immunohistochemical staining showing SphK1 expression in the livers of patients with MASLD (n=7) and healthy individuals (n=7). Scale bar: 20 μm. **B** Representative images of immunohistochemical staining for SphK1 in normal areas and areas adjacent to lipid accumulation (excluding fat vacuoles) in the livers of patients with mild MASLD (n=7). Scale bar: 20 μm. **C** Representative western blot images and quantitative analysis (**D**) of SphK1 and SphK2 expression in the livers of WT mice fed an HFD for 0, 4, 8, or 12 weeks (n = 3 mice/group). The line chart represents the relative intensity of the bands quantified using western blotting. Data are presented as the mean ± SEM. $^{**}p < 0.01$, vs. chow diet-fed mice, unpaired Student's *t* test. **E** Representative western blot images and quantitative analysis (**F**) of SphK1 and SphK2 levels in Hepa1-6 cells treated with palmitic acid (0.6 mM) for 24 h. Data are presented as the mean ± SEM from three independent experiments. $^{**}p < 0.01$, unpaired Student's *t* test.

**Figure S2. Liver damage does not occur in *Sphk2*$^{-/-}$ mice**

**A** H&E staining of liver sections from WT, *Sphk1*$^{-/-}$, and *Sphk2*$^{-/-}$ mice at 4, 8, and 16 weeks of age, respectively. Scale bar: 20 μm. Images are representative of three independent experiments. **B** Serum AST and ALT levels in WT, *Sphk1*$^{-/-}$, and *Sphk2*$^{-/-}$ mice (n=3 per group) were measured using enzymatic kits. **C** Representative flow cytometric images of hepatic monocytes ($Ly6G^-Ly6C^+$), neutrophils ($Ly6G^+Ly6C^+$), and Kupffer cells ($F4/80^+$) first gated on $CD11b^+$ cells. Data are presented as mean ± SEM (n=3–5 per group). $^{**}p < 0.01$, one-way ANOVA. Abbreviations: ALT, alanine transaminase; AST, aspartate aminotransferase; NEU, neutrophils; Mon, monocytes; KCs, Kupffer cells.

**Figure S3. Altered protein profiles of primary hepatocytes isolated from *Sphk2*$^{-/-}$ mice**

**A** Principal component analysis (PCA) of primary hepatocytes isolated from WT and *Sphk2*$^{-/-}$ mice (n = 3 mice/group; 8 weeks of age) showed good reproducibility for each biological replicate. **B** The number of DEPs whose expression increased or decreased in the absence of SphK2 (left panel), and a volcano plot illustrating significantly differentially expressed proteins in primary hepatocytes isolated from WT and *Sphk2*$^{-/-}$ mice. Abbreviation: DEPs, differentially expressed proteins.

**Figure S4. Impaired vesicle transport in the hepatocytes of *Sphk2*$^{-/-}$ mice**

**A** Representative western blot images showing MTP, PDI, Sar1a, and Sar1b expression in the livers of WT and *Sphk2*$^{-/-}$ mice (n = 3 mice/group). **B, C** Top 20 enriched GO terms for DEPs in terms of cellular components and molecular functions. Abbreviations: MTP, microsomal triglyceride transfer protein; PDI, protein disulfide isomerase; Sar1a, secretion-associated Ras-related 1a; Sar1b, secretion-associated Ras-related 1b.

**Figure S5. Posttranscriptional regulation of the SNARE complex by SphK2 in hepatocytes**

The mRNA levels of *Stx5a*, *Gs28*, and *Sec22b* were measured in Hepa1-6 cells treated with ABC294640 for 0, 24, 48, and 72 h using quantitative real-time PCR. Error bars indicate mean ± SEM from three independent experiments, one-way ANOVA.

**Figure S6. Positive controls for the treatments of MG132, 3-MA and $NH_4Cl$**

**A** Hepa1-6 cells were treated with MG132 (10 μM), leading to an increase in ubiquitination, as detected by immunoblotting with anti-K48 ubiquitin antibodies. Images are representative of three independent experiments. **B** Western blot analysis of LC3BI and LC3BII protein levels

in Hepa1-6 cells treated with 3-MA (2.5 mM). Images are representative of three independent experiments. **C** Representative immunofluorescence microscopy images showing Hoechst 33342 and LysoTracker Red staining in Hepa1-6 cells, with or without $NH_4Cl$ treatment (8 mM). Scale bar: 20 μm. Images are representative of three independent experiments.

**Figure S7. SphK2 regulates SNARE stability and accelerates SNARE degradation in a lysosome-dependent manner**

**A** Representative Western blot images showing the protein expression levels of SphK2 in Hepa1-6 cells after treatment with siRNA. Images are representative of three independent experiments. **B** The mRNA levels of *Stx5a*, *Gs28*, and *Sec22b* were measured in Hepa1-6 cells with or without *Sphk2* knockdown using quantitative real-time PCR. Error bars indicate mean ± SEM from three independent experiments, unpaired Student's *t-test*. **C** Western blot analysis was performed on equal amounts of cell lysates from Hepa1-6 cells with or without *Sphk2* knockdown following treatment with cycloheximide (CHX, 5 mg/mL) for various durations. Membranes were probed with antibodies against STX5A, GS28, and SEC22B, and the corresponding quantitative graphs are shown in panel **D**. Error bars indicate the mean ± SEM from three independent experiments. *$p < 0.05$, **$p < 0.01$, and ***$p < 0.001$, unpaired Student's $t$ test. **E** Representative Western blot images showing the levels of STX5A, GS28, and SEC22B in whole-cell lysates from Hepa1-6 cells with or without *Sphk2* knockdown, treated individually with the inhibitors $NH_4Cl$ (8 mM), 3-MA (2.5 mM), or MG132 (10 μM) for 24 hours. Images represent at least three independent experiments. **F** Representative images and quantitative analysis of Oil Red O staining in Hepa1-6 cells untreated or treated with *Sphk2* knockdown or $NH_4Cl$. Scale bar: 10 μm. Error bars indicate the mean ± SEM from three independent experiments. *$p < 0.05$ and ***$p < 0.001$, one-way ANOVA. **G** Intracellular TG levels were measured in Hepa1-6 cells untreated or treated with *Sphk2* knockdown or $NH_4Cl$.

Error bars indicate mean ± SEM of five independent experiments. *$p < 0.05$, one-way ANOVA.

**Figure S8. SphK2 has minimal regulatory effects on macroautophagy**

**A** Gene set enrichment analysis revealed that the gene sets involved in "lysosome" and "lysosome organization" were enriched in primary hepatocytes isolated from *Sphk2*$^{-/-}$ mice. **B** Representative western blot images showing Beclin1, p-Beclin1, p62, and LC3B expression in Hepa1-6 cells treated with ABC294640 for 0, 24, 48, and 72 h. **C** Representative western blot images showing the phosphorylated and total levels of ULK1 and Beclin1, as well as the levels of p62, LC3BI, and LC3BII. **D** LC3BI and LC3BII levels in Hepa1-6 cells treated with ABC294640 or BafA1 (30 nM) were determined using western blotting. Images are representative of three independent experiments.

**Figure S9. HSC70 knockdown reverses the lipid accumulation induced by ABC294640 treatment**

**A** Representative Western blot images of LAMP2A, HSC70, and ATG5 after knockdown of their respective genes using siRNA. Images are representative of three independent experiments. **B, C** Oil Red O staining and quantification of Hepa1-6 cells after *Atg5* and *Hsc70* knockdown. Notably, after *Hsc70* knockdown, the accumulation of lipid droplets in Hepa1-6 cells progressed more slowly than that in *Atg5* knockdown cells. Scale bar: 10 μm. Error bars indicate the mean ± SEM from six independent experiments. **$p < 0.01$ and ***$p < 0.001$, one-way ANOVA. **D** Intracellular and (**E**) secreted TG levels in Hepa1-6 cells before and after *Atg5* and *Hsc70* knockdown, respectively. Error bars indicate the mean ± SEM from four independent experiments. *$p < 0.05$ and **$p < 0.01$, one-way ANOVA. **F** The KFERQ sequences identified in STX5A, GS28, and SEC22B by the "KFERQ Finder" tool are highlighted in different colors and indicate specific types of post-translational modifications as

follows: **Yellow:** Canonical motifs within standard motifs; **Blue:** Phosphorylation-activated motifs within standard motifs; **Green:** Acetylation-activated motifs within standard motifs; **Purple:** Motifs activated by both acetylation and phosphorylation within advanced motifs.

**Figure S10. *Sphk2* deficiency does not affect mitochondrial function**

**A** Primary hepatocytes from WT and *Sphk2*$^{-/-}$ mice (n=3 per group) were isolated and seeded in triplicate into the wells of an extracellular flux analyzer for the mitochondrial stress test. The oxygen consumption rate (OCR) was measured over time (min) using a Seahorse XF-24e Extracellular Flux Analyzer. Oligomycin (Oligo, a complex V inhibitor, 1 μM), FCCP (a protonophore, 0.5 μM), and antimycin A (AntiA, a complex III inhibitor, 0.5 μM) were sequentially added to analyze ATP-linked respiration, proton leak, maximal respiratory capacity, mitochondrial reserve capacity, and non-mitochondrial respiration. **B** The extracellular acidification rate (ECAR) was measured using a glycolysis stress test. Glucose (10 mM), oligomycin (1 μM), and 2-deoxyglucose (2-DG, 50 mM) were added sequentially. **C** Representative flow cytometric analysis of JC-1 staining in Hepa1-6 cells treated with ABC294640 or CCCP (positive control). **D** Statistical analysis of JC-1 monomer fluorescence in Hepa1-6 cells treated with ABC294640. Error bars indicate the mean ± SEM from three independent experiments, unpaired Student's *t-test*.

**Figure S11. SphK2 knockdown exerts an inhibitory effect on the mTORC2 pathway**

Representative western blots were performed to determine the levels of phosphorylated and total AKT, GSK3β, SGK1, and PKCα proteins in Hepa1-6 cells treated with *Sphk2* knockdown. Images are representative of three independent experiments.

**Figure S12. S1P improves intracellular lipid accumulation independent of S1PR**

**activation**

**A** Representative images of Oil Red O staining and (**B**) quantification of the intracellular lipid content in Hepa1-6 cells treated with or without ABC294640 (30 μM), S1P (2 μM), BAF312 (100 μM) or CYM5520 (10 μM). Scale bar: 10 μm. Data are presented as mean ± SEM from three independent experiments; ***$p < 0.001$, one-way ANOVA.

**Supplementary Table S1. Clinical characteristics of normal and MASLD livers**

| | Normal liver | MASLD liver | *p*-value |
|---|---|---|---|
| ALT (U/L) | 27 ± 11.34 | 59.50 ± 34.27 | 0.026* |
| AST (U/L) | 42.5 ± 33.45 | 45.71 ± 15.21 | 0.921 |
| GGT (U/L) | 59.14 ± 52.92 | 76.33 ± 36.57 | 0.669 |
| ALP (U/L) | 104.63 ± 52.12 | 80.71 ± 16.56 | 0.045* |
| TBIL (μmol/L) | 13.638.05 | 14.754.53 | 0.595 |
| DBIL (μmol/L) | 5.882.95 | 5.00 ± 1.20 | 0.081 |
| IBIL (μmol/L) | 7.75 ± 5.60 | 9.75 ± 3.62 | 0.933 |
| TC (mmol/L) | 3.71 ± 1.02 | 5.55 ± 1.48 | 0.015* |
| TG (mmol/L) | 1.03 ± 0.34 | 3.02 ± 1.54 | 0.011* |
| TP (g/L) | 59.91 ± 6.39 | 80.46 ± 6.00 | <0.0001*** |
| Albumin (g/L) | 32.11 ± 6.47 | 47.19 ± 5.48 | <0.0001*** |
| Ig (g/L) | 27.80 ± 4.92 | 33.28 ± 3.40 | 0.005** |
| GLU (mmol/L) | 8.68 ± 6.82 | 6.511.77 | 0.252 |
| Insulin (pmol/L) | 115.9 ± 137.89 | 134.28 ± 39.16 | 0.644 |
| C peptide (pmol/L) | 870.55 ± 781.28 | 1180.33 ± 131.98 | 0.417 |

Note: All values are presented as mean ± SEM. Statistical analysis was conducted using the Student's *t* test to compare the two groups. *$p < 0.05$; **$p < 0.01$; and ***$p < 0.001$. Abbreviations: MASLD, ALT, alanine aminotransferase; AST, aspartate aminotransferase; GGT, γ-glutamyl transferase; ALP, alkaline phosphatase; TBIL, total bilirubin; DBIL, direct bilirubin; IBIL, indirect bilirubin; TC, total cholesterol; TG, triglyceride; TP, total protein; Ig, immunoglobulin; GLU, glucose.

**Supplementary Table S2. List of primary antibodies and reagents used for western blotting, immunohistochemistry, and immunofluorescence staining**

| Antibody | Source | Identifier |
|---|---|---|
| Anti-SphK1 | Proteintech | 10670-1-AP |
| Anti-SphK2 | Proteintech | 17096-1-AP |
| Anti-SphK2 | Santa Cruz Biotechnology | Sc-517192 |
| Anti-ACC1 | Boster | BM4414 |
| Anti-SREBP1 | ZEN-BIOSCIENCE | 347061 |
| Anti-CD36 | Boster | PB9371 |
| Anti-PNPLA3 | Proteintech | 11442-1-AP |
| Anti-FAS | Boster | PB9865 |
| Anti-LXRα | ZEN-BIOSCIENCE | R381851 |
| Anti-SCD1 | Proteintech | 28678-1-AP |
| Anti-PGC1α | ZEN-BIOSCIENCE | 381615 |
| Anti-LCADα | ZEN-BIOSCIENCE | R23690 |
| Anti-ACADM | ZEN-BIOSCIENCE | R26384 |
| Anti-ACOX1 | ZEN-BIOSCIENCE | E23371 |
| Anti-β-actin | Proteintech | 81115-1-AP |
| Anti-SEC31 | ABclonal | A9321 |
| Anti-SEC24 | ABclonal | A10797 |
| Anti-SEC23 | Boster | A05287-2 |
| Anti-SEC13 | Affinity | DF12734 |
| Anti-SEC22B | Abcam | Ab181076 |

| | | |
|---|---|---|
| Anti-STX5A | Proteintech | 26711-1-AP |
| Anti-GS28 | Santa Cruz Biotechnology | Sc-133148 |
| Anti-LAMP2A | Abcam | ab125068 |
| Anti-LAMP2A | Abways | CY5518 |
| Anti-HSC70 | ABclonal | A2487 |
| Anti-pAKT$^{Ser473}$ | Proteintech | 66444-1-Ig |
| Anti-AKT | Proteintech | 10176-2-AP |
| Anti-pGSK3β | Proteintech | 67558-1-Ig |
| Anti-GSK3β | Proteintech | 22104-1-AP |
| Anti-PKCα$^{Ser657}$ | ZEN-BIOSCIENCE | R26336 |
| Anti-PKCα | ZEN-BIOSCIENCE | R25382 |
| Anti-SGK1$^{Ser422}$ | Affinity | AF3001 |
| Anti-SGK1 | ZEN-BIOSCIENCE | R381169 |
| Anti-Raptor | ZEN-BIOSCIENCE | 381155 |
| Anti-Rictor | Proteintech | 27248-1-AP |
| Anti-MTP | Boster | A01715-1 |
| Anti-PDI | Cell Signaling Technology | C81H6 |
| Anti-Sar1a | FineTest | FNab07601 |
| Anti-Sar1b | FineTest | FNab07603 |
| Anti-GAPDH | Proteintech | 60004-1-Ig |
| Anti-pBeclin1$^{Ser15}$ | ABclonal | AP1252 |
| Anti-Beclin1 | ABclonal | A7353 |
| Anti-p62 | ABclonal | A19700 |
| Anti-LC3B | Boster | BM4827 |
| Anti-ERGIC | ABclonal | A10440 |

| Regents | Source | Identifier |
| --- | --- | --- |
| ER-Tracker Red | Beyotime Biotechnology | C1041-1 |
| Golgi-Tracker Red | Beyotime Biotechnology | C1043-1 |
| Hoechst | Beyotime Biotechnology | C1028 |
| BODIPY$^{TM}$ 493/503 | Thermo Fisher Scientific | D3922 |
| Lyso-Tracker Red | Beyotime Biotechnology | C1046 |

**Supplementary Table S3. List of primers for quantitative real-time polymerase chain reaction**

| Gene | Forward primer (5′ - 3′) | Reserve primer (5′ - 3′) |
| --- | --- | --- |
| *Sec22b* | CTGACGATGATCGCCCGTG | TGCTTAGCCTGACTCTGATACTG |
| *Gs28* | AAATGGATCAAGCCAAGACAGAA | TTGTCGTTTACTCCTGTAAGCC |
| *Stx5a* | CTGTCCCCTGCAACTGCTATC | CAGGCAGACTGGAACTCCTG |

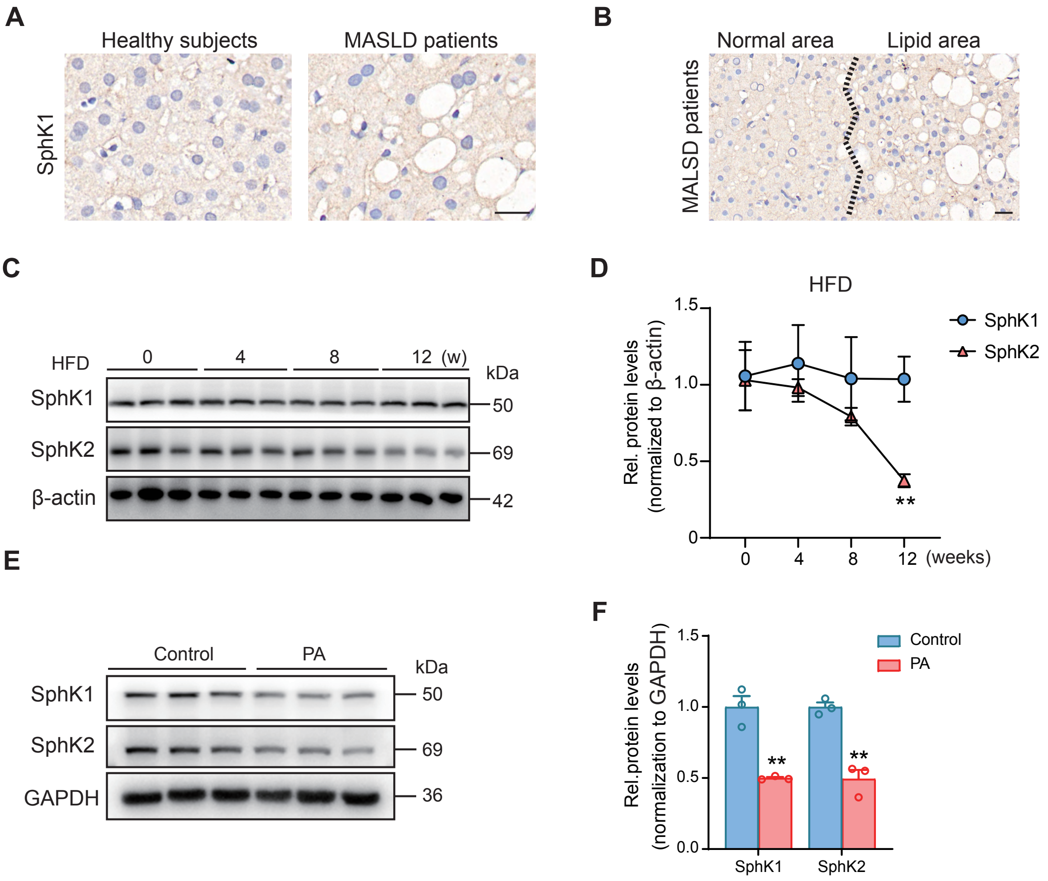
A
Healthy subjects
MASLD patients
SphK1
B
Normal area
Lipid area
MALSD patients
C
HFD
0
4
8
12 (w)
kDa
SphK1
SphK2
β-actin
50
69
42
D
HFD
Rel. protein levels (normalized to β-actin)
SphK1
SphK2
**
0
4
8
12 (weeks)
E
Control
PA
kDa
SphK1
SphK2
GAPDH
50
69
36
F
Rel.protein levels (normalization to GAPDH)
Control
PA
**
**
SphK1
SphK2

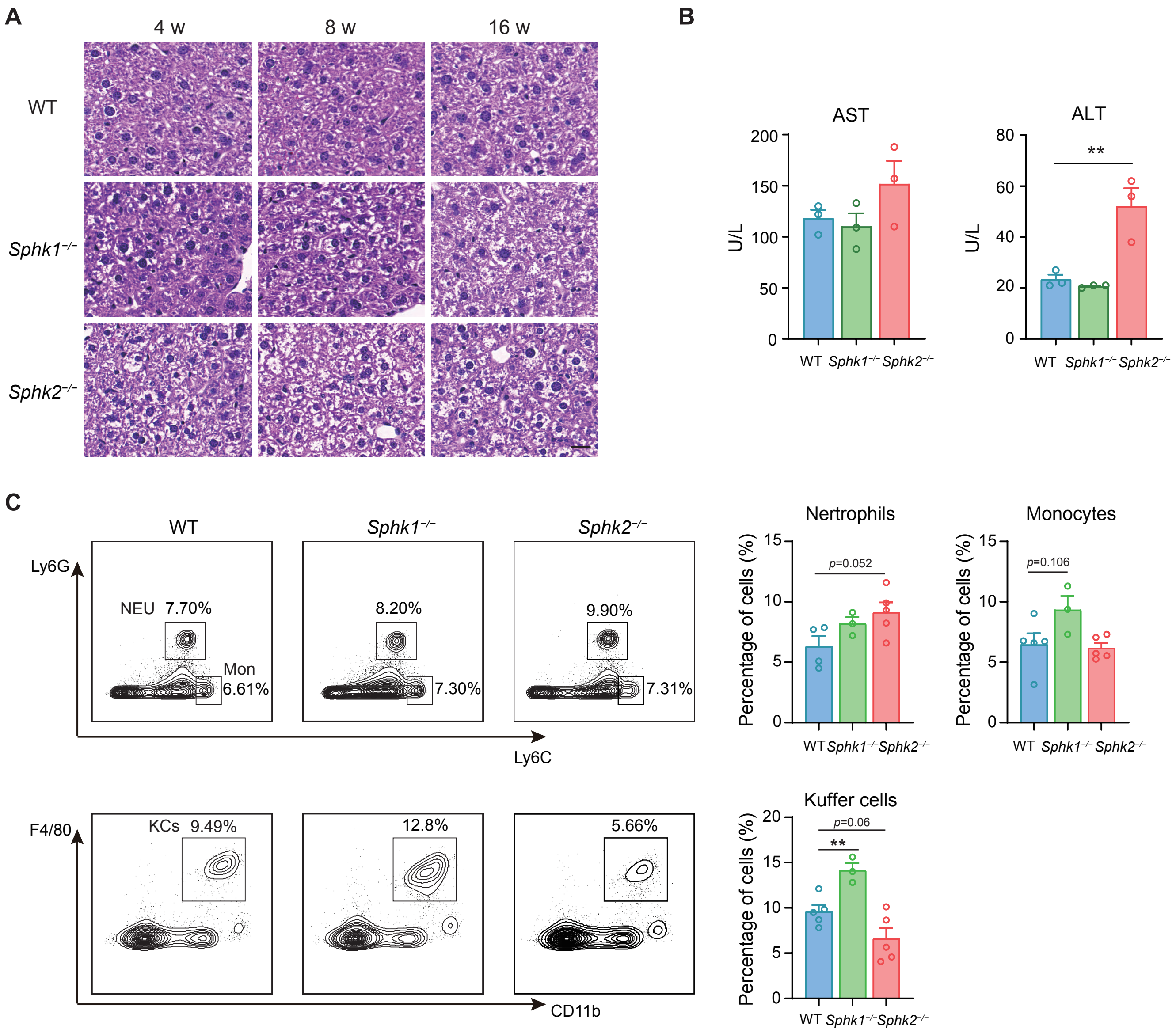
A
4 w
8 w
16 w
WT
Sphk1−/−
Sphk2−/−
B
AST
ALT
U/L
**
WT Sphk1−/− Sphk2−/−
C
WT
Sphk1−/−
Sphk2−/−
Ly6G
NEU 7.70%
Mon
6.61%
8.20%
7.30%
9.90%
7.31%
Ly6C
Nertrophils
Monocytes
Percentage of cells (%)
p=0.052
p=0.106
F4/80
KCs 9.49%
12.8%
5.66%
CD11b
Kuffer cells
p=0.06
**

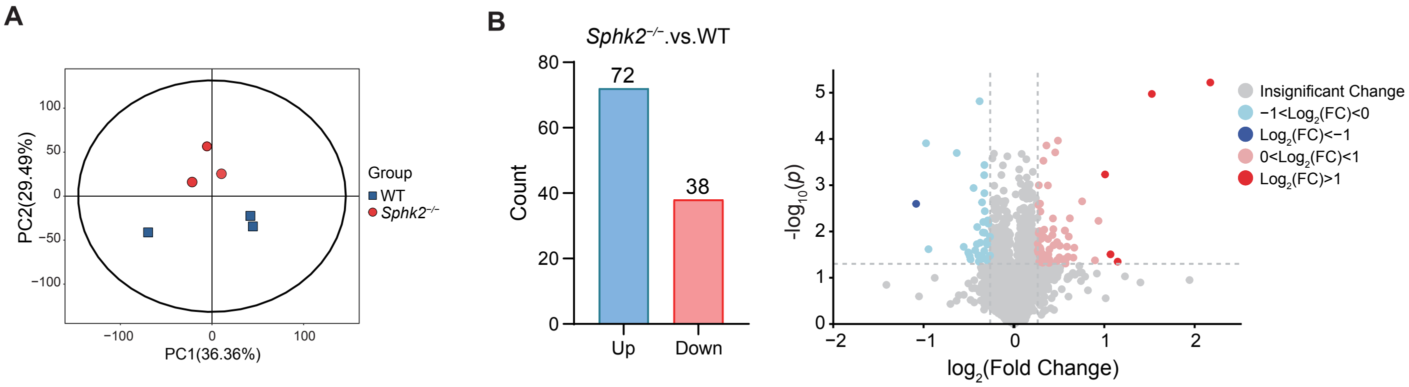
A
B
PC2(29.49%)
PC1(36.36%)
100
50
0
−50
−100
Group
WT
Sphk2−/−
Sphk2−/−.vs.WT
Count
80
60
40
20
72
38
Up
Down
-log10(p)
log2(Fold Change)
Insignificant Change
−1<Log2(FC)<0
Log2(FC)<−1
0<Log2(FC)<1
Log2(FC)>1

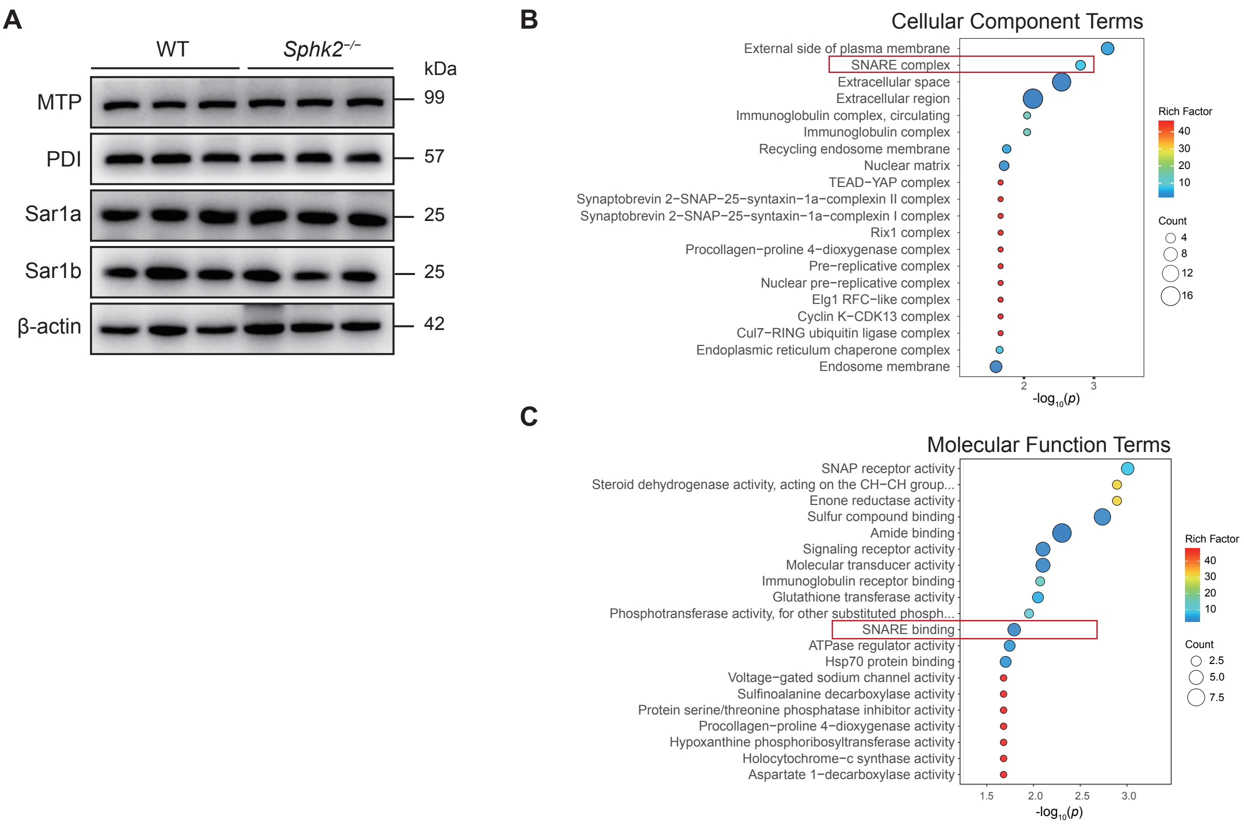
A
WT
Sphk2−/−
kDa
MTP
99
PDI
57
Sar1a
25
Sar1b
25
β-actin
42
B
Cellular Component Terms
External side of plasma membrane
SNARE complex
Extracellular space
Extracellular region
Immunoglobulin complex, circulating
Immunoglobulin complex
Recycling endosome membrane
Nuclear matrix
TEAD−YAP complex
Synaptobrevin 2−SNAP−25−syntaxin−1a−complexin II complex
Synaptobrevin 2−SNAP−25−syntaxin−1a−complexin I complex
Rix1 complex
Procollagen−proline 4−dioxygenase complex
Pre−replicative complex
Nuclear pre−replicative complex
Elg1 RFC−like complex
Cyclin K−CDK13 complex
Cul7−RING ubiquitin ligase complex
Endoplasmic reticulum chaperone complex
Endosome membrane
2
3
-log10(p)
Rich Factor
40
30
20
10
Count
4
8
12
16
C
Molecular Function Terms
SNAP receptor activity
Steroid dehydrogenase activity, acting on the CH−CH group...
Enone reductase activity
Sulfur compound binding
Amide binding
Signaling receptor activity
Molecular transducer activity
Immunoglobulin receptor binding
Glutathione transferase activity
Phosphotransferase activity, for other substituted phosph...
SNARE binding
ATPase regulator activity
Hsp70 protein binding
Voltage−gated sodium channel activity
Sulfinoalanine decarboxylase activity
Protein serine/threonine phosphatase inhibitor activity
Procollagen−proline 4−dioxygenase activity
Hypoxanthine phosphoribosyltransferase activity
Holocytochrome−c synthase activity
Aspartate 1−decarboxylase activity
1.5
2.0
2.5
3.0
-log10(p)
Rich Factor
40
30
20
10
Count
2.5
5.0
7.5

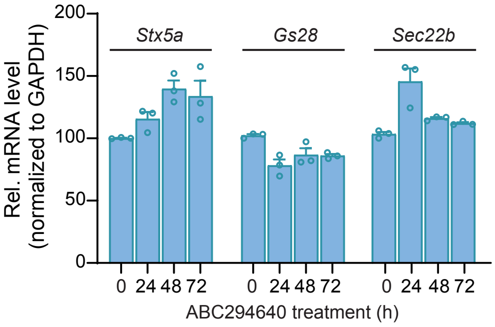

Stx5a
Gs28
Sec22b
Rel. mRNA level
(normalized to GAPDH)
200
150
100
50
0
0 24 48 72
0 24 48 72
0 24 48 72
ABC294640 treatment (h)

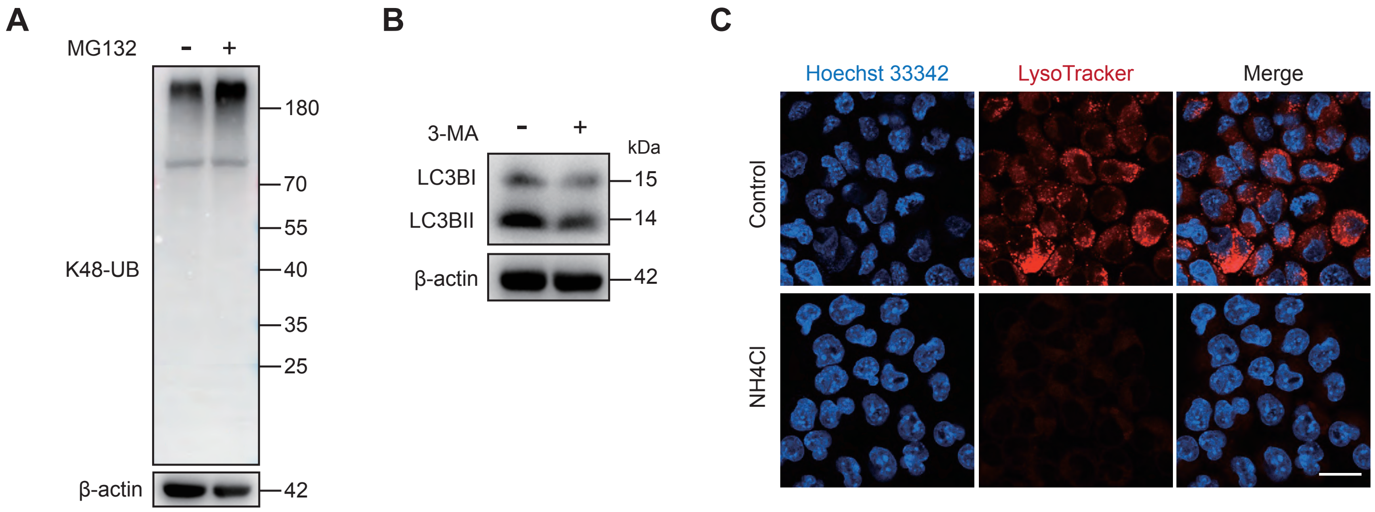
A
MG132 - +
K48-UB
180
70
55
40
35
25
β-actin
42
B
3-MA - +
kDa
LC3BI
LC3BII
15
14
β-actin
42
C
Hoechst 33342
LysoTracker
Merge
Control
NH4Cl

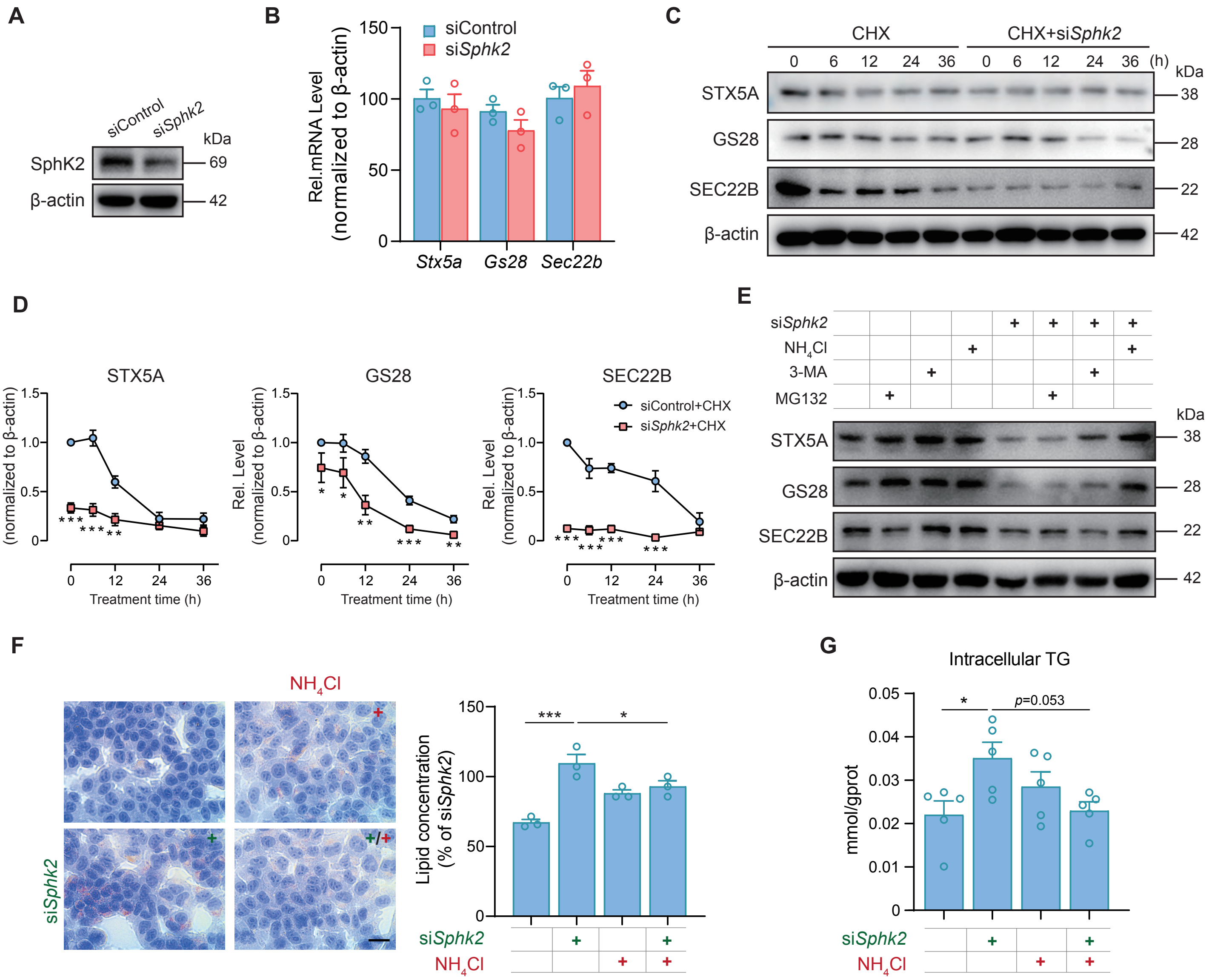

A
siControl
siSphk2
kDa
SphK2
69
β-actin
42
B
siControl
siSphk2
Rel.mRNA Level
(normalized to β-actin)
Stx5a
Gs28
Sec22b
C
CHX
CHX+siSphk2
0 6 12 24 36 (h)
kDa
STX5A
38
GS28
28
SEC22B
22
β-actin
42
D
STX5A
GS28
SEC22B
siControl+CHX
siSphk2+CHX
Rel. Level
(normalized to β-actin)
Treatment time (h)
E
siSphk2
NH4Cl
3-MA
MG132
kDa
STX5A
38
GS28
28
SEC22B
22
β-actin
42
F
NH4Cl
siSphk2
Lipid concentration
(% of siSphk2)
siSphk2
NH4Cl
G
Intracellular TG
p=0.053
mmol/gprot
siSphk2
NH4Cl

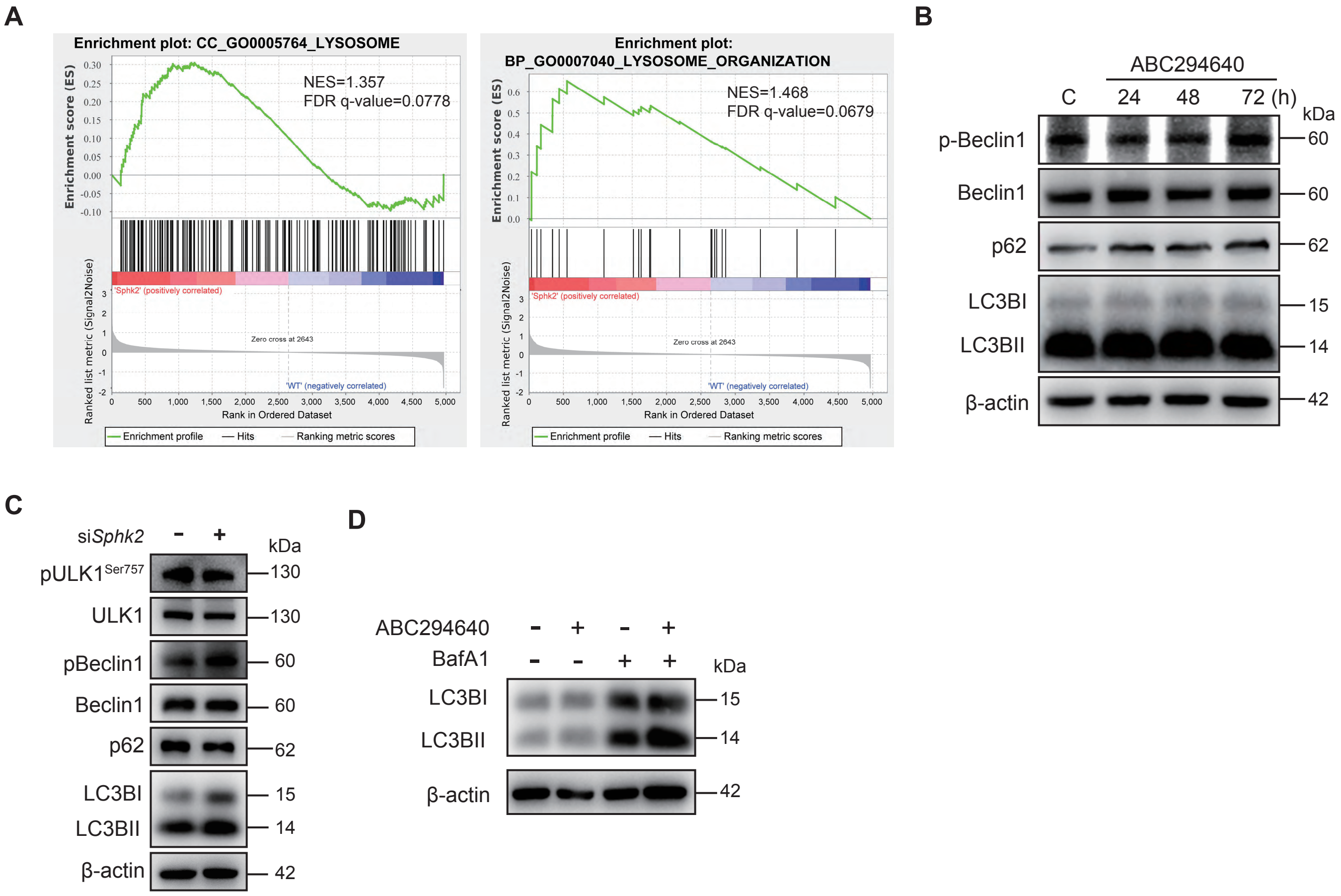
A
Enrichment plot: CC_GO0005764_LYSOSOME
NES=1.357
FDR q-value=0.0778
Enrichment score (ES)
Ranked list metric (Signal2Noise)
'Sphk2' (positively correlated)
Zero cross at 2643
'WT' (negatively correlated)
Rank in Ordered Dataset
Enrichment profile
Hits
Ranking metric scores
Enrichment plot:
BP_GO0007040_LYSOSOME_ORGANIZATION
NES=1.468
FDR q-value=0.0679
B
ABC294640
C
24
48
72 (h)
kDa
p-Beclin1
Beclin1
p62
LC3BI
LC3BII
β-actin
60
60
62
15
14
42
C
siSphk2
-
+
kDa
pULK1Ser757
ULK1
pBeclin1
Beclin1
p62
LC3BI
LC3BII
β-actin
130
130
60
60
62
15
14
42
D
ABC294640
BafA1
kDa
LC3BI
LC3BII
β-actin
15
14
42

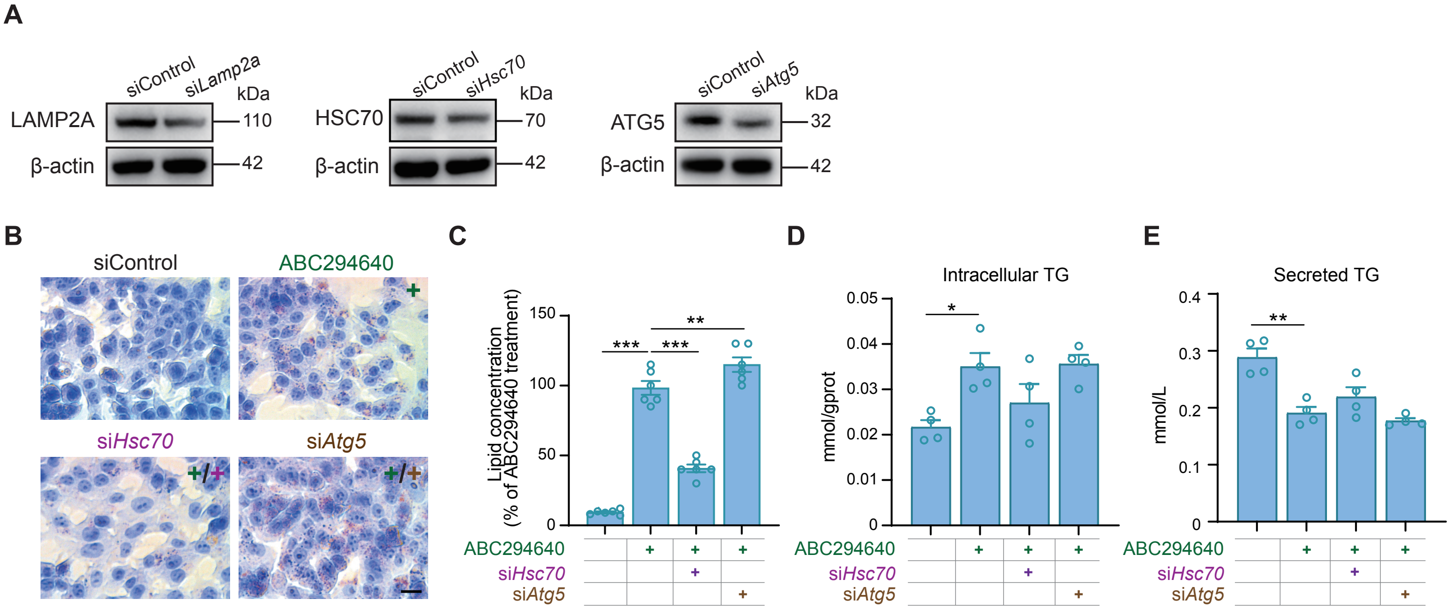


F

UniProt ID:Q8K1E0 protein names: Syntaxin-5
MIPRKRYGSKNTDQGVYLGLSKTQVLSPATAISSSSDSTPLPTPVALVPSPPDTMSCRDRTQEFQSACKSLQSRQNGIQTSKPALHAARQ
CSEFTLMARRIGKDLSNTFA**KLEKL**TILA**KRKSL**FDDKAVEIEELT**YIIKQ**DINSLNKQIAQL**QDFVR**AKGSQSGRHLQTHSNTIVVSLQ
SKLASMSNDFKSVLEVRTENLKQQRNRREQFSRAPVSALPLAPNNLGGGPIILGAESRASRDVAIDMMDPRTSQQLQLIDEQDSYIQSRA
DTMQNIESTIVELGSIFQQLAHMVKEQEETIQRIDENVLGAQLDVEAAHSEI**LKYFQ**SVTSNRWLMVKIFLILIVFFI IFVVFLA

UniProt ID: O88630 protein names: Golgi SNAP receptor complex member 1 (28 kDa Golgi SNARE protein) (28 kDa cis-Golgi SNARE p28) (GOS-28)
MAAGTSNYWE**DLRKQ**ARQLENELDLKLVSFSKLCTSYSHSGSRDGGRDRYSSDTTPLLNGSSQDRMFETMAIEIEQLLARLTGVNDKM
AEYTHSAGVPSLNAALMHTLQRH**RDILQ**DYTHEFHKTKANFTAIRERENLMGSVRKDIESYKSGSGVNNRRTELFLKEHDHLRNSDRLI
EETISIAMATKENMTSQRGMLKSIHSK MNTLANRFPAVNSLIQRINLRKRRDSLILGGVIGICTILLLLYAFH

UniProt ID: O08547 protein names: Vesicle-trafficking protein SEC22b (ER-Golgi SNARE of 24 kDa) (ERS-24) (ERS24) (SEC22 vesicle-trafficking protein homolog B) (SEC22 vesicle-trafficking protein-like 1)(mSec22b)
MVLLTMIARVADGLPLAASMQEDEQSGRDLQQYQSQAKQLFRKLNEQSPTRCTLEAGAMTFHYIIEQGVCYLVLCEAAFPKKLAFAYL
EDLHSEFDEQHGKKVPTVSRPYSFIEFDTFIQ**KTKKLYI**DSRARRNLGSINTELQDVQRIMVANIEEVLQRGEALSALDSKANNLSSLSKK
YRQDAKYLNMRSTYAKLAAVAVFFIMLIV YVRFWWL

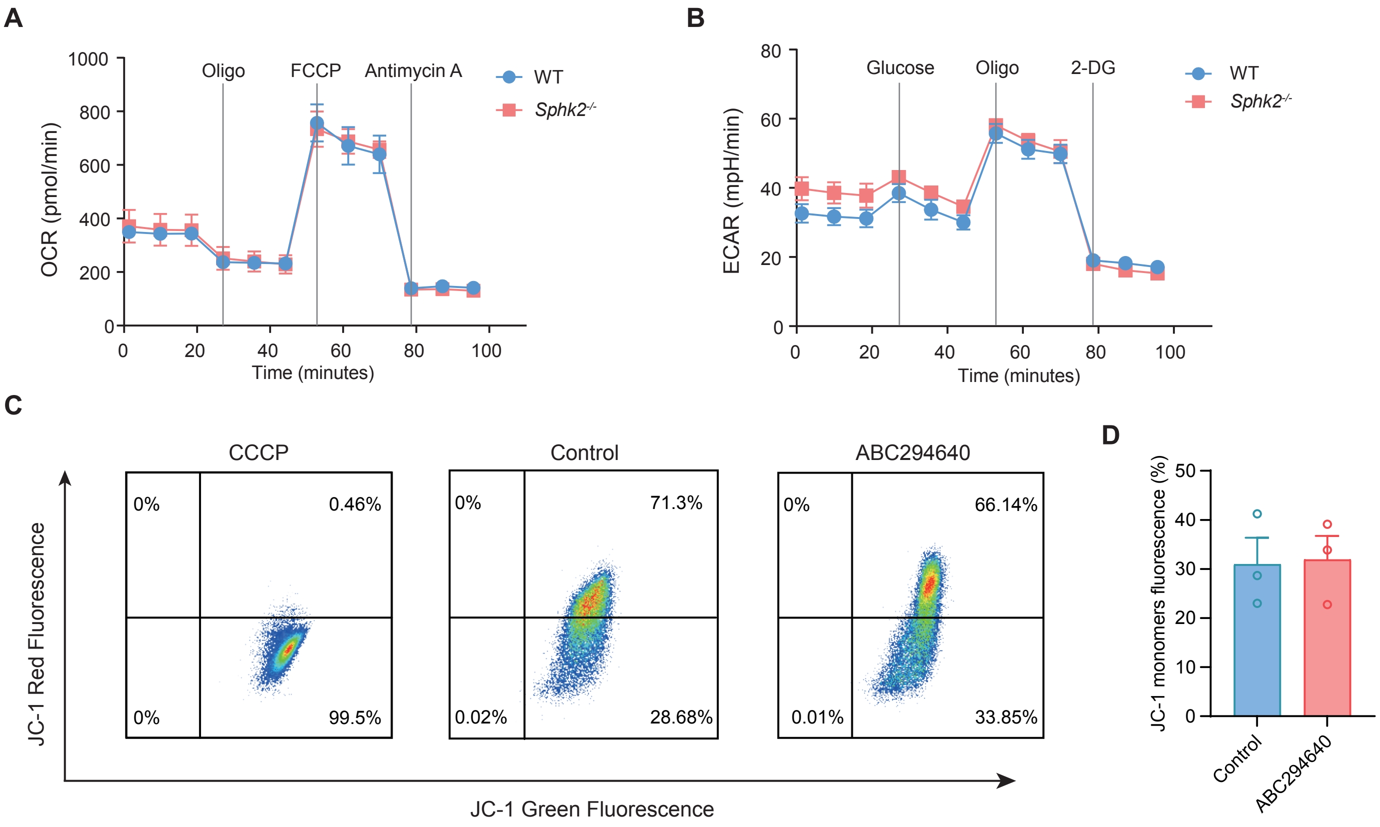
A
1000
800
600
400
200
0
OCR (pmol/min)
0
20
40
60
80
100
Time (minutes)
Oligo
FCCP
Antimycin A
WT
Sphk2-/-
B
80
60
40
20
0
ECAR (mpH/min)
0
20
40
60
80
100
Time (minutes)
Glucose
Oligo
2-DG
WT
Sphk2-/-
C
CCCP
Control
ABC294640
0%
0.46%
0%
99.5%
0%
71.3%
0.02%
28.68%
0%
66.14%
0.01%
33.85%
JC-1 Red Fluorescence
JC-1 Green Fluorescence
D
50
40
30
20
10
0
JC-1 monomers fluorescence (%)
Control
ABC294640

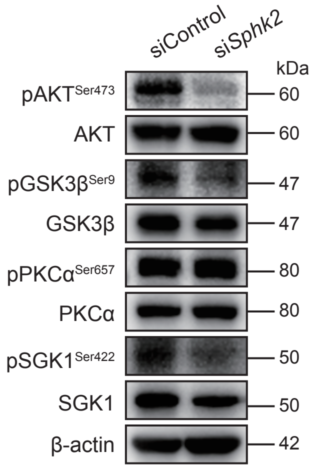
siControl
siSphk2
kDa
pAKT^Ser473
AKT
pGSK3β^Ser9
GSK3β
pPKCα^Ser657
PKCα
pSGK1^Ser422
SGK1
β-actin
60
60
47
47
80
80
50
50
42

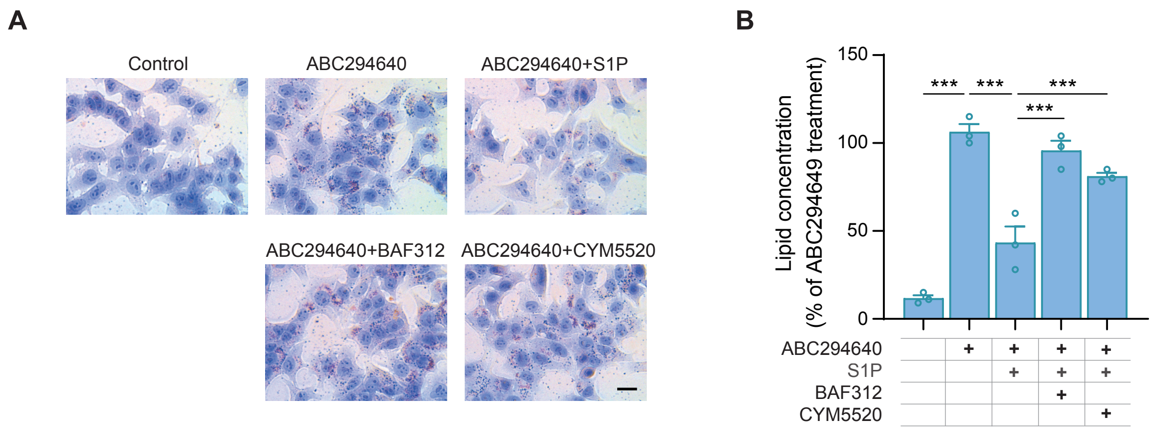

A
Control
ABC294640
ABC294640+S1P
ABC294640+BAF312
ABC294640+CYM5520
B
Lipid concentration
(% of ABC294649 treatment)
150
100
50
0
***
***
***
***
ABC294640
S1P
BAF312
CYM5520
