# Supplementary material for: Sphingosine kinase 2 deficiency impairs VLDL secretion by inhibiting mTORC2 phosphorylation and activating chaperone-mediated autophagy
Source: Cell Death Differ. 2025 Apr 8;32(10):1886–99. doi: 10.1038/s41418-025-01507-6 (PMC12500862; doi:10.1038/s41418-025-01507-6)
Supplement: Supplementary file 2 — Original Data Files [file 41418_2025_1507_MOESM2_ESM.pdf]

Fig. 2F

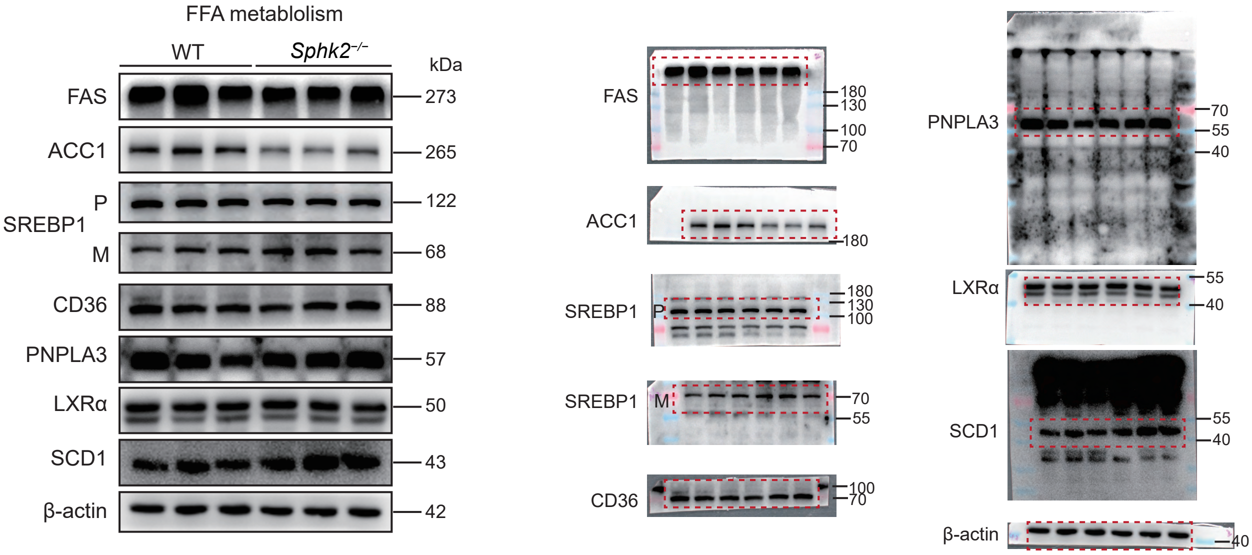
FFA metablolism
WT
Sphk2−/−
kDa
FAS
273
ACC1
265
SREBP1
P
122
M
68
CD36
88
PNPLA3
57
LXRα
50
SCD1
43
β-actin
42
FAS
180
130
100
70
ACC1
180
SREBP1
P
180
130
100
SREBP1
M
70
55
CD36
100
70
PNPLA3
70
55
40
LXRα
55
40
SCD1
55
40
β-actin
40

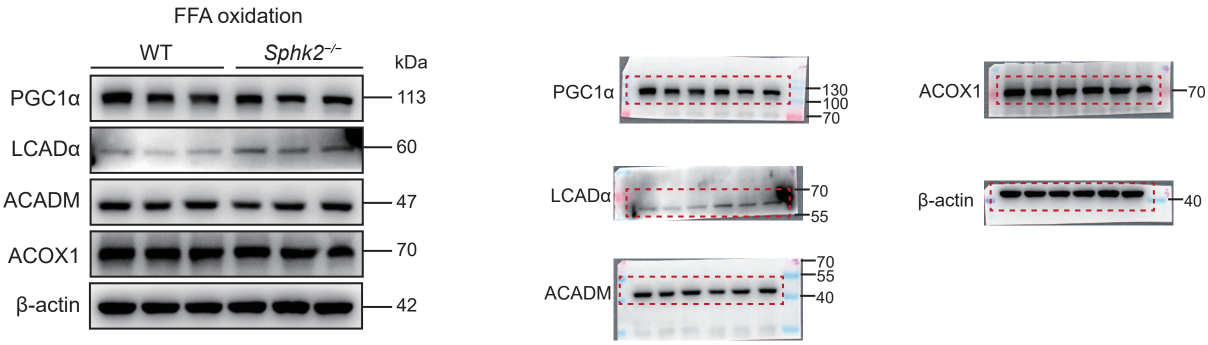
FFA oxidation
WT
Sphk2−/−
kDa
PGC1α
113
LCADα
60
ACADM
47
ACOX1
70
β-actin
42
PGC1α
130
100
70
LCADα
70
55
ACADM
70
55
40
ACOX1
70
β-actin
40

Fig. 3C

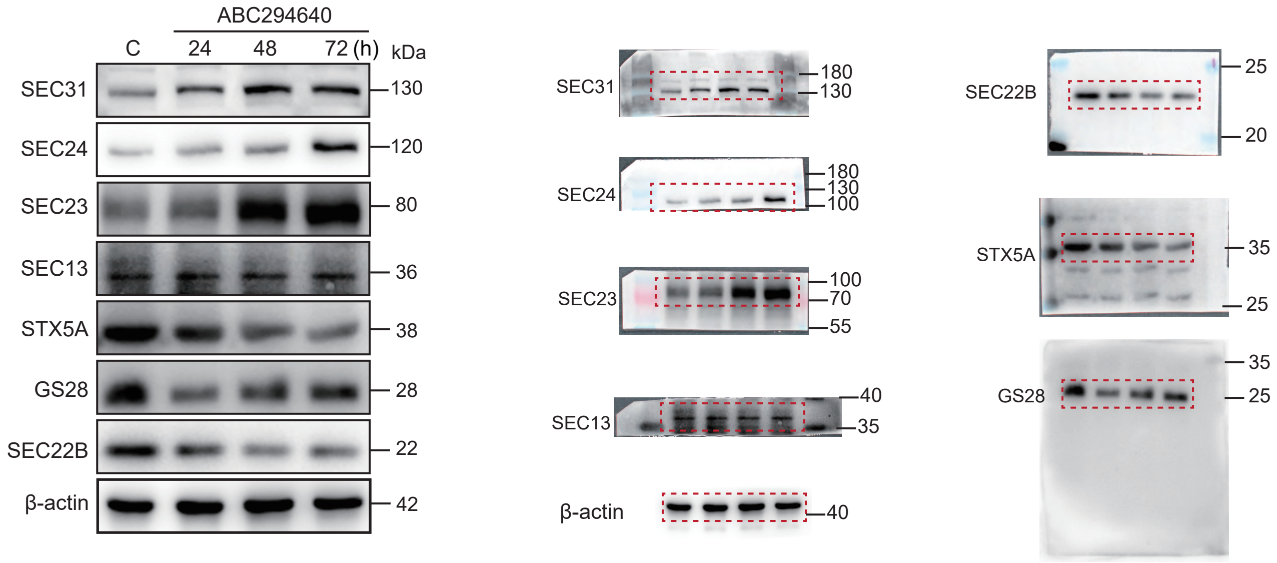
ABC294640
C
24
48
72 (h)
kDa
SEC31
SEC24
SEC23
SEC13
STX5A
GS28
SEC22B
β-actin
130
120
80
36
38
28
22
42
180
130
100
70
55
40
35
25
20

Fig. 3E

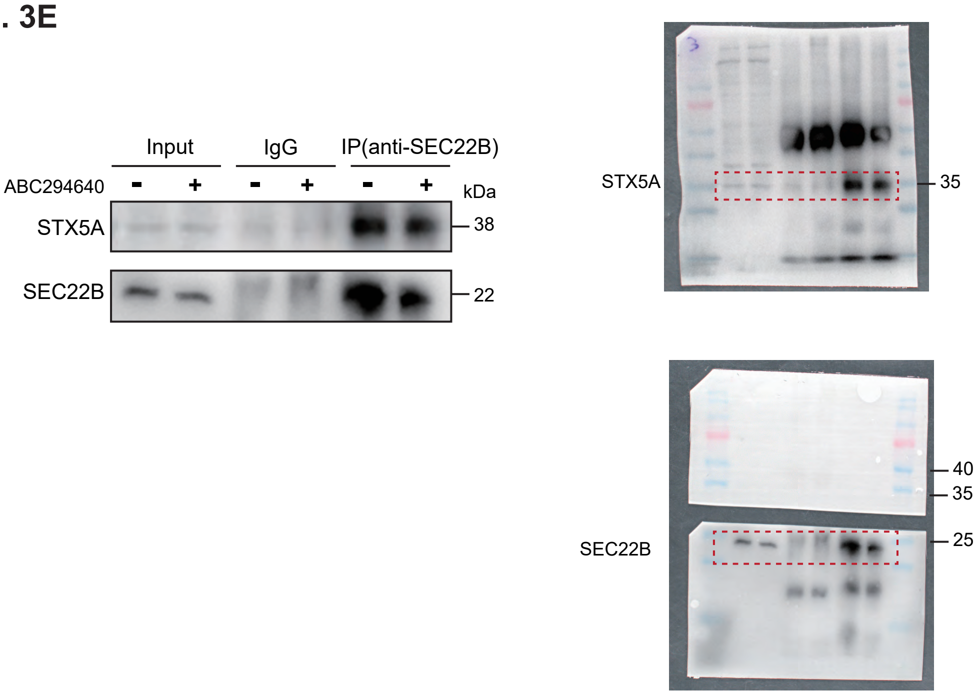
Input
IgG
IP(anti-SEC22B)
ABC294640
- + - + - +
kDa
STX5A
SEC22B
38
22
35
40
25

Fig. 4A

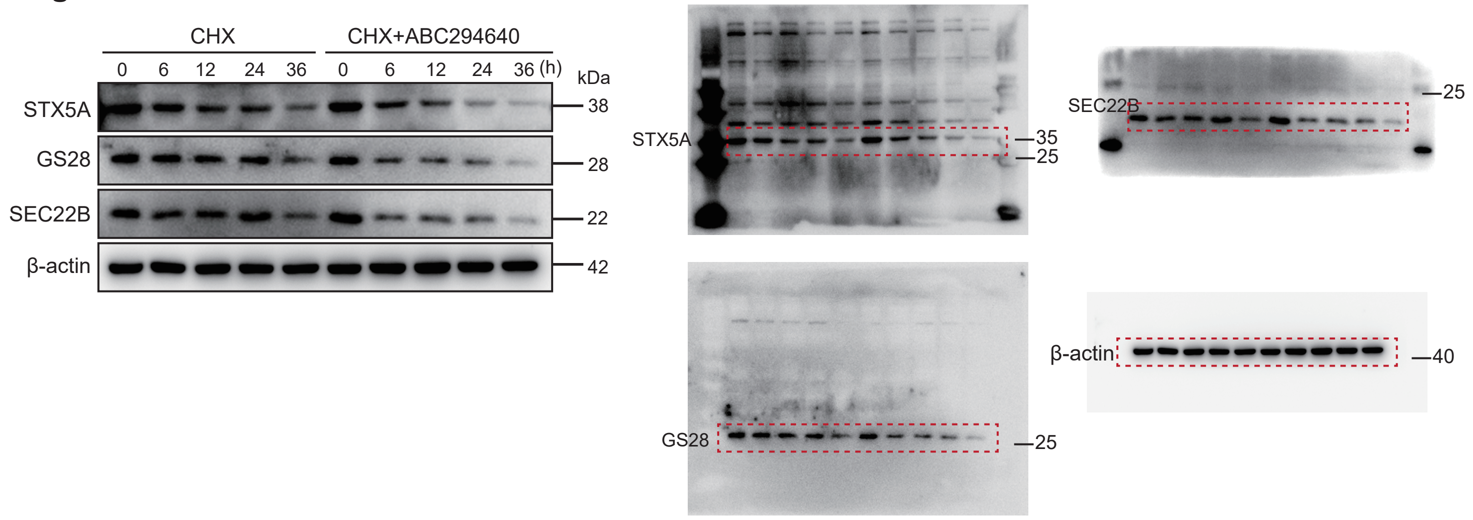
CHX
CHX+ABC294640
0 6 12 24 36 0 6 12 24 36 (h)
kDa
STX5A
GS28
SEC22B
β-actin
38
28
22
42
STX5A
35
25
SEC22B
25
GS28
25
β-actin
40

Fig. 4C

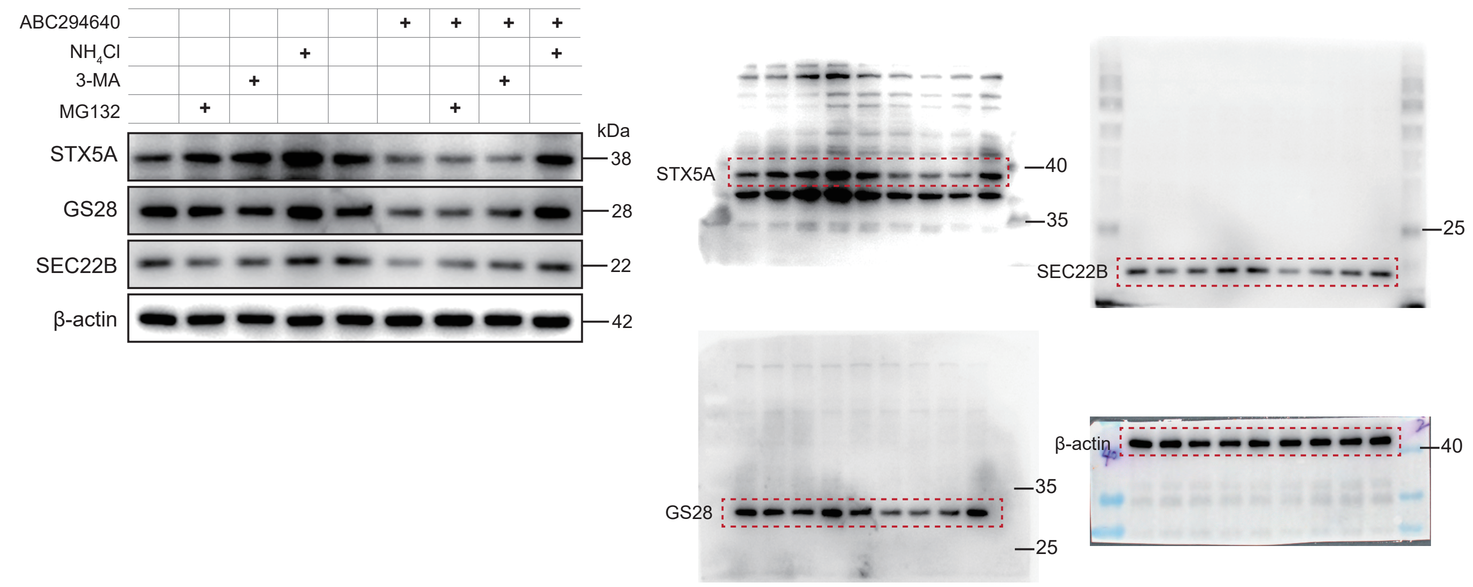
ABC294640
NH4Cl
3-MA
MG132
kDa
STX5A
GS28
SEC22B
β-actin
38
28
22
42
STX5A
40
35
SEC22B
25
GS28
35
25
β-actin
40

Fig. 5C

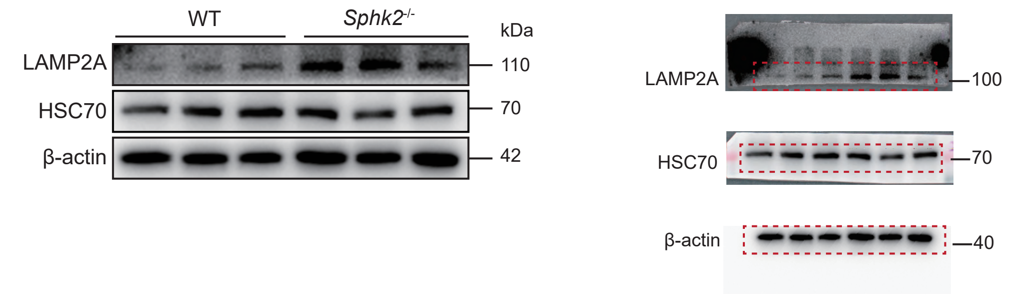

Fig. 5D

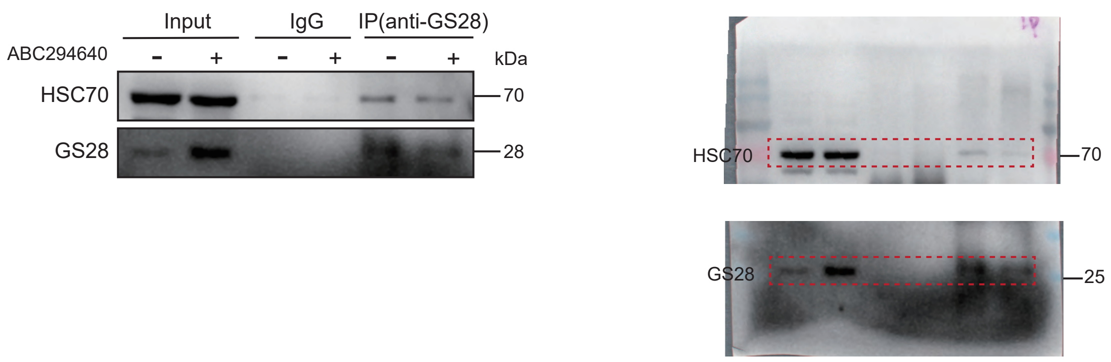

Fig. 5J

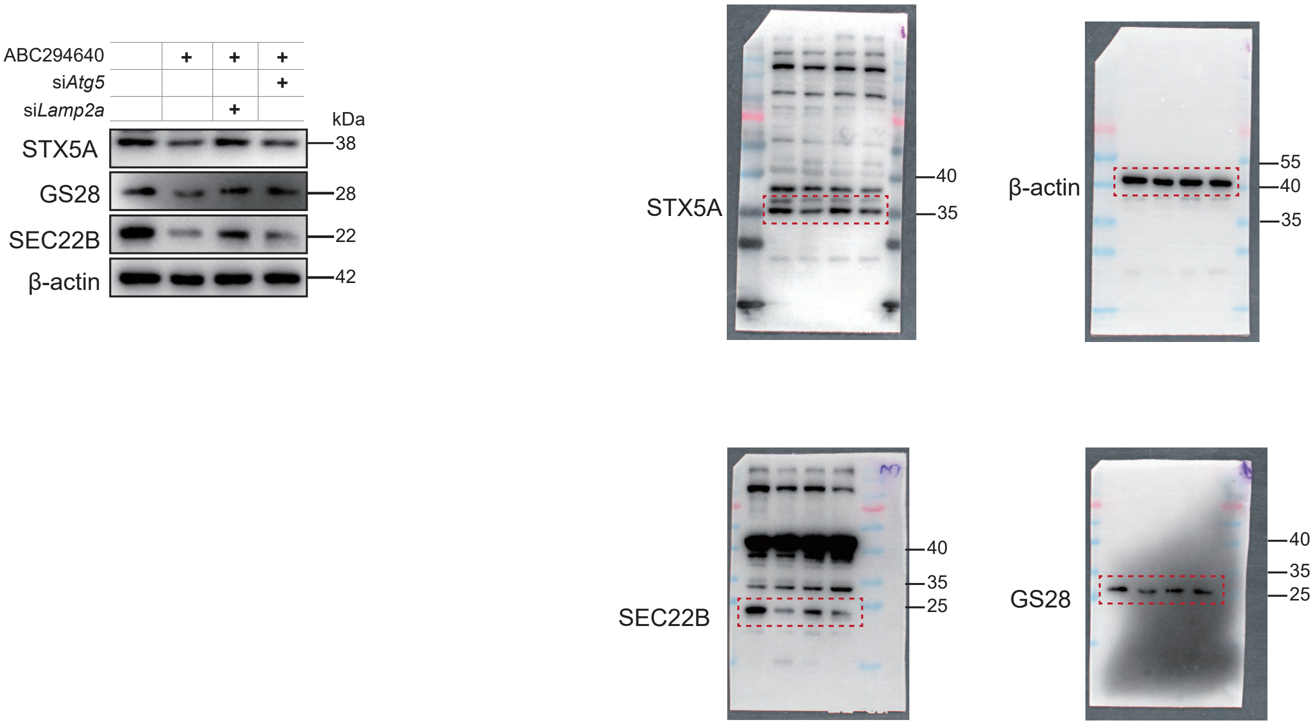

Fig. 6E

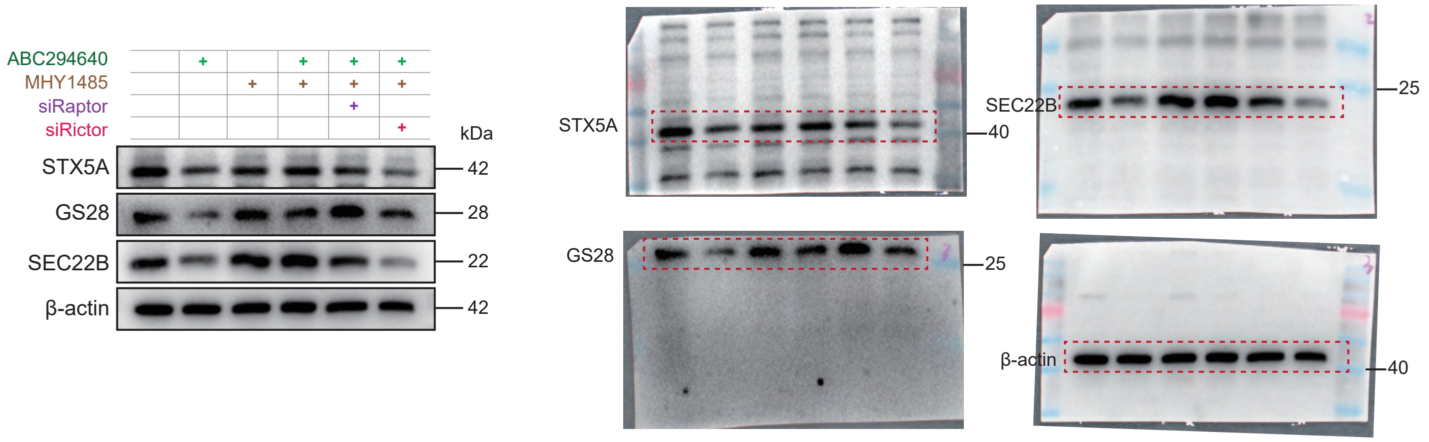
ABC294640
MHY1485
siRaptor
siRictor
kDa
STX5A
GS28
SEC22B
β-actin
42
28
22
42
STX5A
40
SEC22B
25
GS28
25
β-actin
40

Fig. 6F

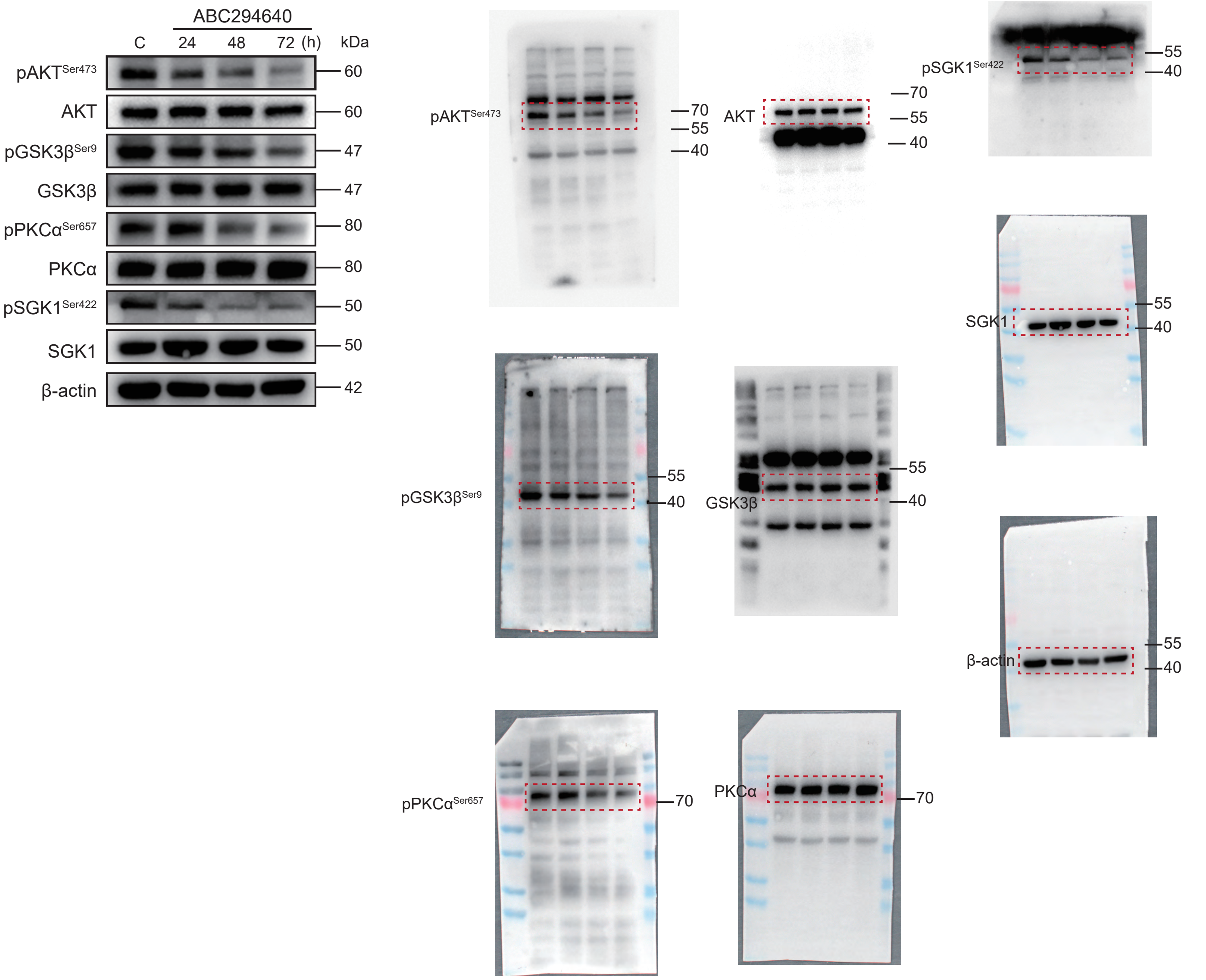
ABC294640
C
24
48
72 (h)
kDa
pAKTSer473
AKT
pGSK3βSer9
GSK3β
pPKCαSer657
PKCα
pSGK1Ser422
SGK1
β-actin
60
60
47
47
80
80
50
50
42
pAKTSer473
70
55
40
AKT
70
55
40
pSGK1Ser422
55
40
SGK1
55
40
pGSK3βSer9
55
40
GSK3β
55
40
β-actin
55
40
pPKCαSer657
70
PKCα
70

Fig. 7G

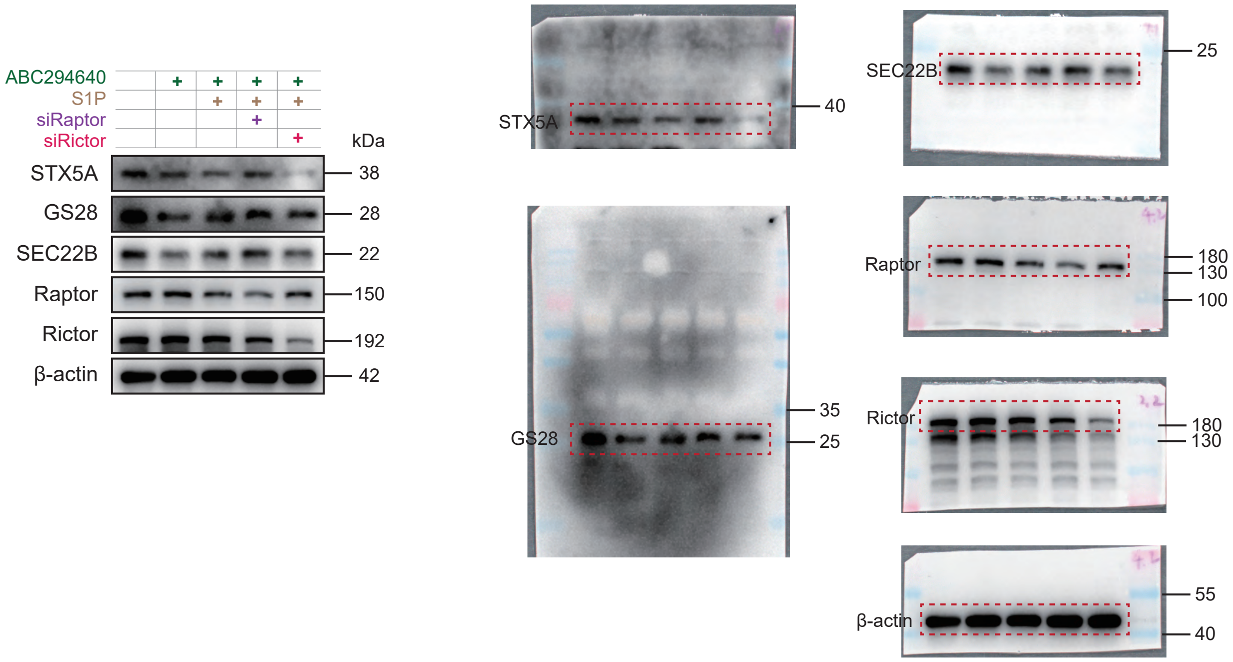

ABC294640
S1P
siRaptor
siRictor
kDa
STX5A
38
GS28
28
SEC22B
22
Raptor
150
Rictor
192
β-actin
42
40
35
25
25
180
130
100
180
130
55
40

Fig. 7I

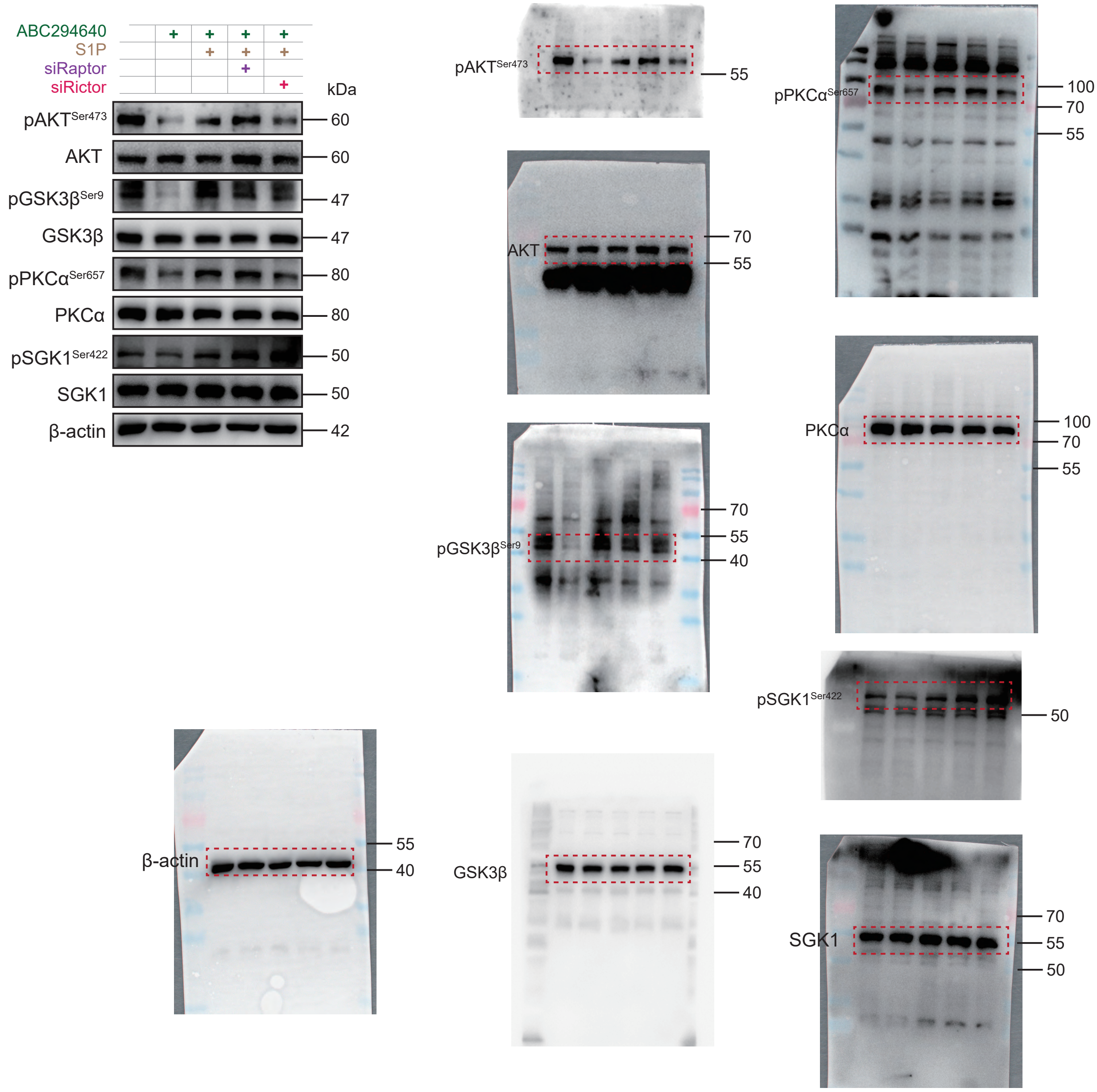

ABC294640
S1P
siRaptor
siRictor
kDa
pAKT[Ser473]
60
AKT
60
pGSK3β[Ser9]
47
GSK3β
47
pPKCα[Ser657]
80
PKCα
80
pSGK1[Ser422]
50
SGK1
50
β-actin
42
55
70
55
70
55
40
100
70
55
100
70
55
50
55
40
70
55
40
70
55
50

Fig.S1C

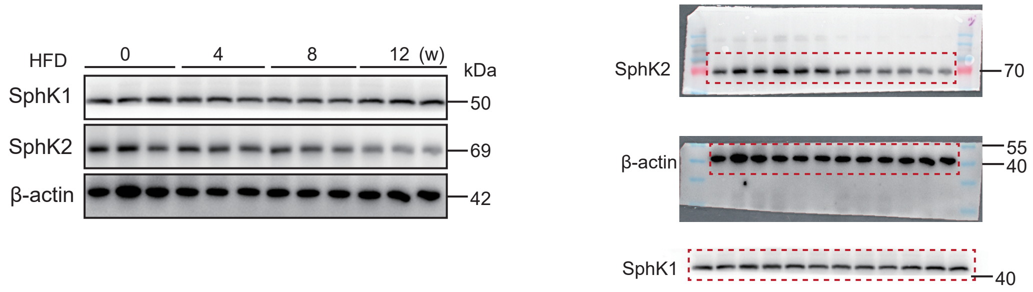

Fig. S1E

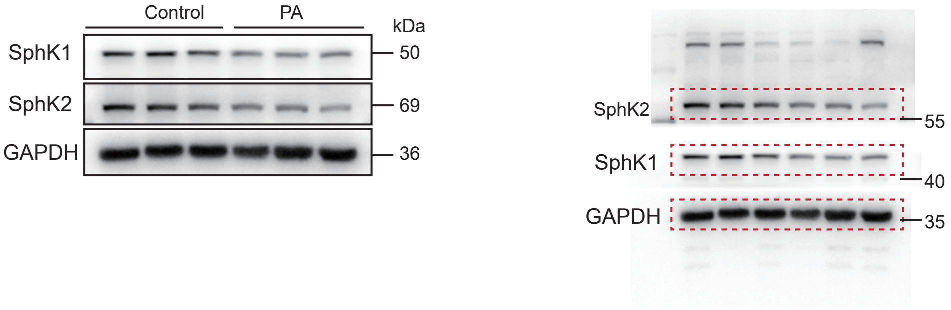

Fig. S4A

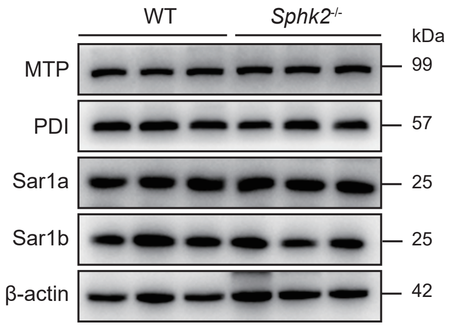

WT
Sphk2-/-
kDa
MTP
99
PDI
57
Sar1a
25
Sar1b
25
β-actin
42

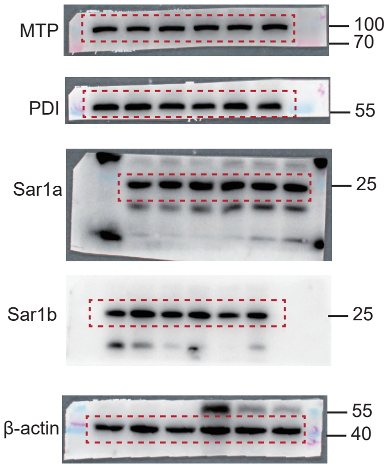

MTP
100
70
PDI
55
Sar1a
25
Sar1b
25
β-actin
55
40

Fig. S6A

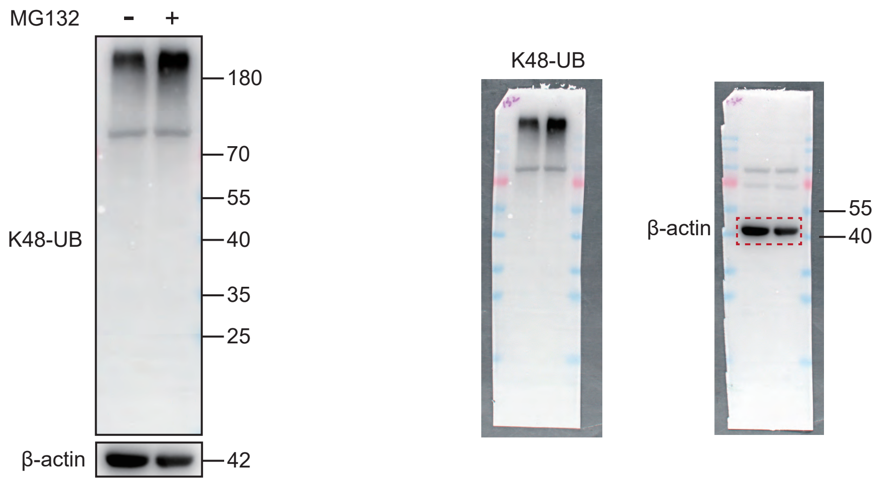

Fig. S6B

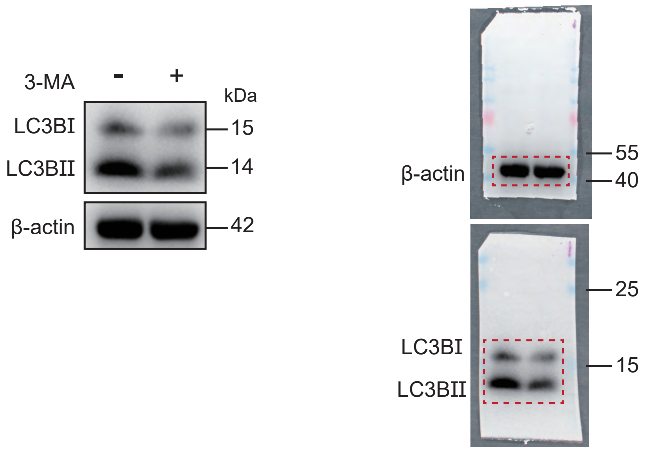

Fig. S7A

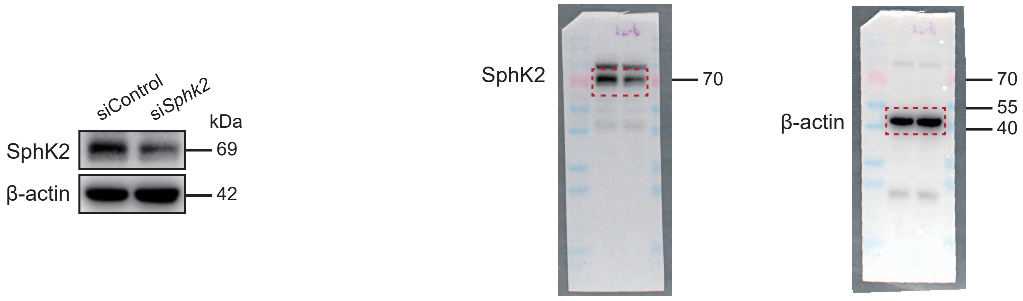

Fig. S7C

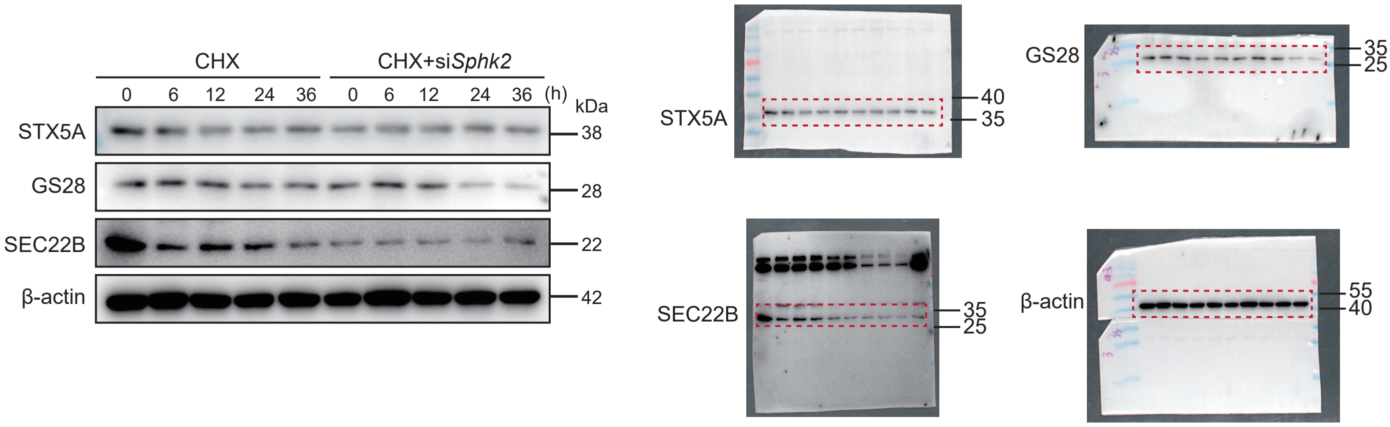

Fig. S7E

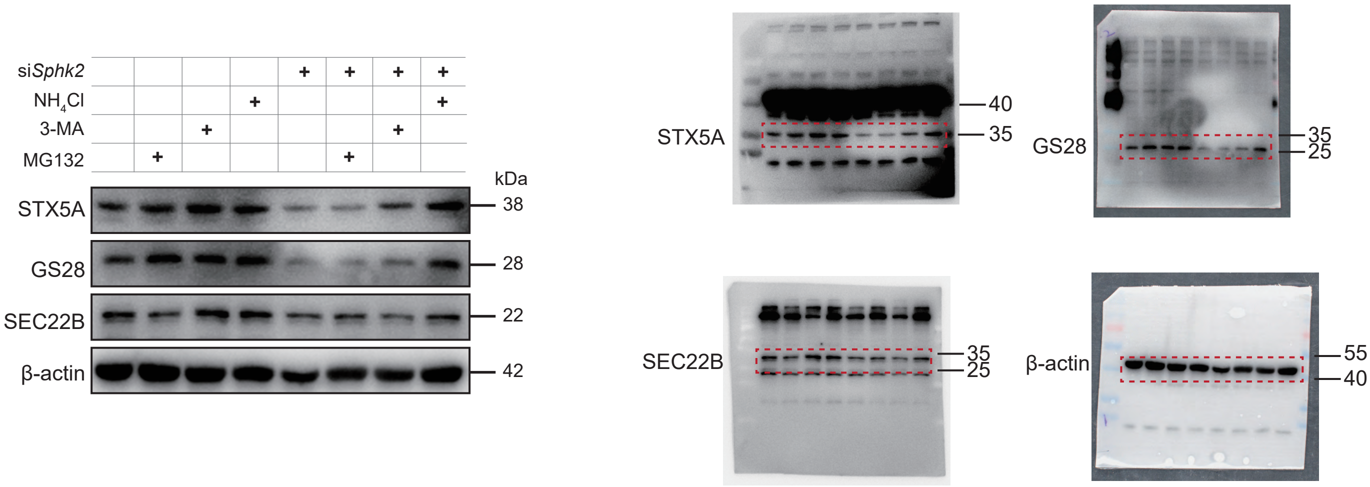

Fig. S8B

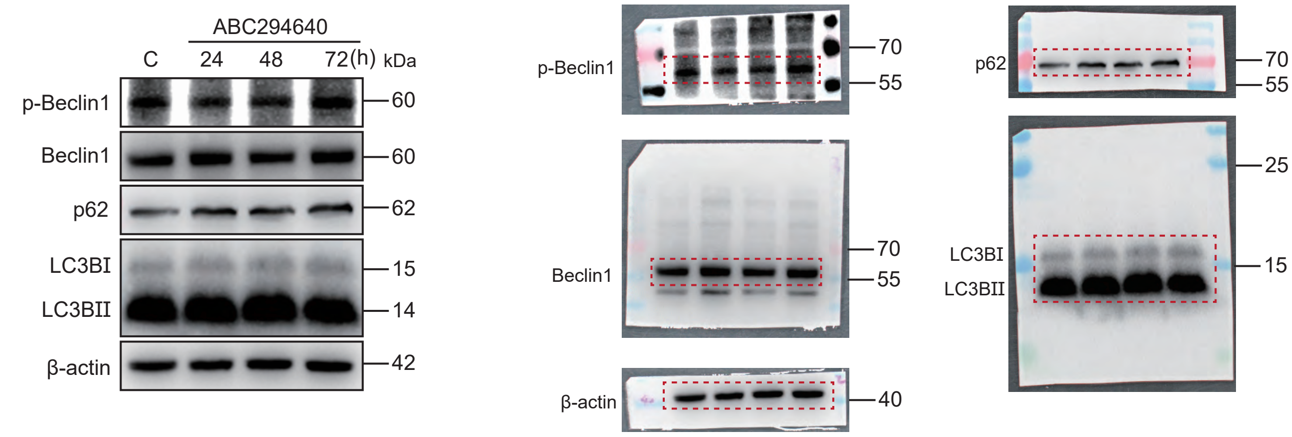

Fig. S8C

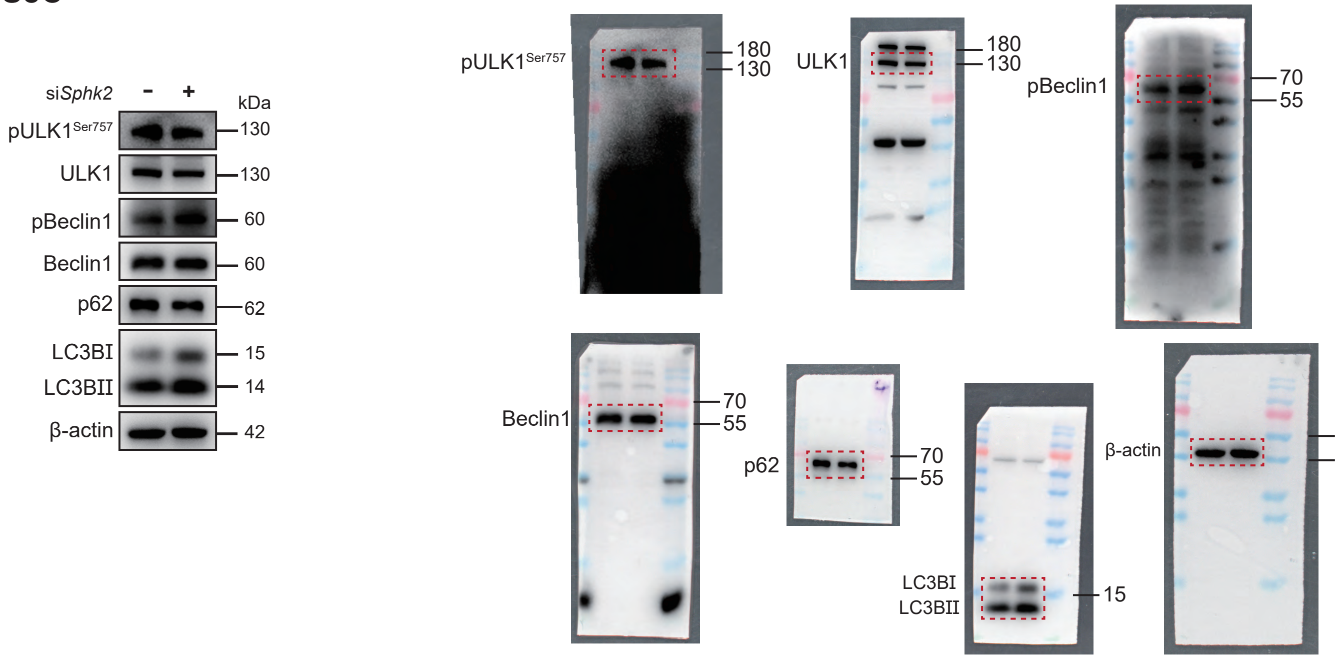

Fig. S8D

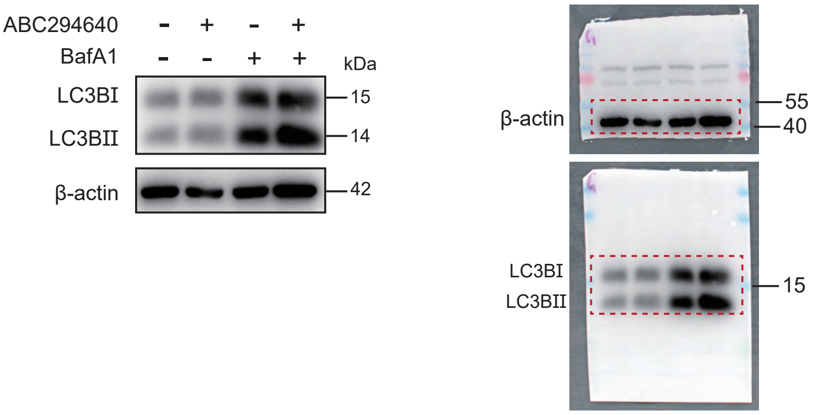

Fig. S9A

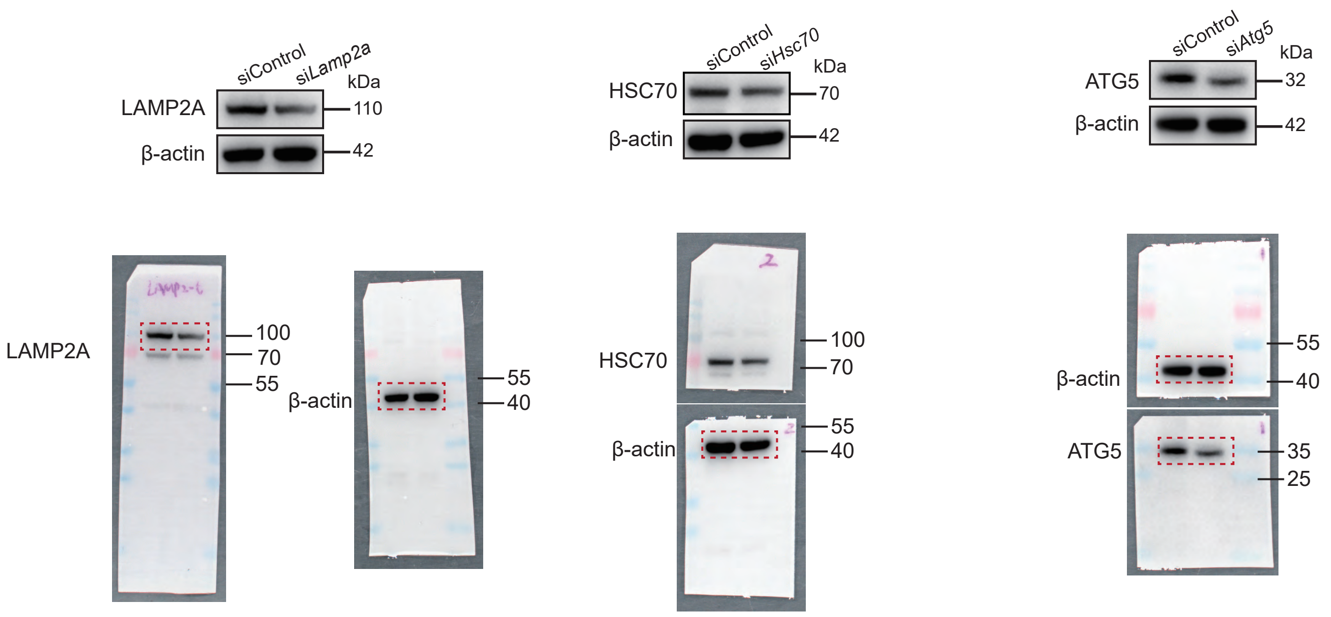
siControl
siLamp2a
kDa
LAMP2A
110
β-actin
42
siControl
siHsc70
kDa
HSC70
70
β-actin
42
siControl
siAtg5
kDa
ATG5
32
β-actin
42
LAMP2A
100
70
55
β-actin
55
40
HSC70
100
70
β-actin
55
40
β-actin
55
40
ATG5
35
25

Fig. S11

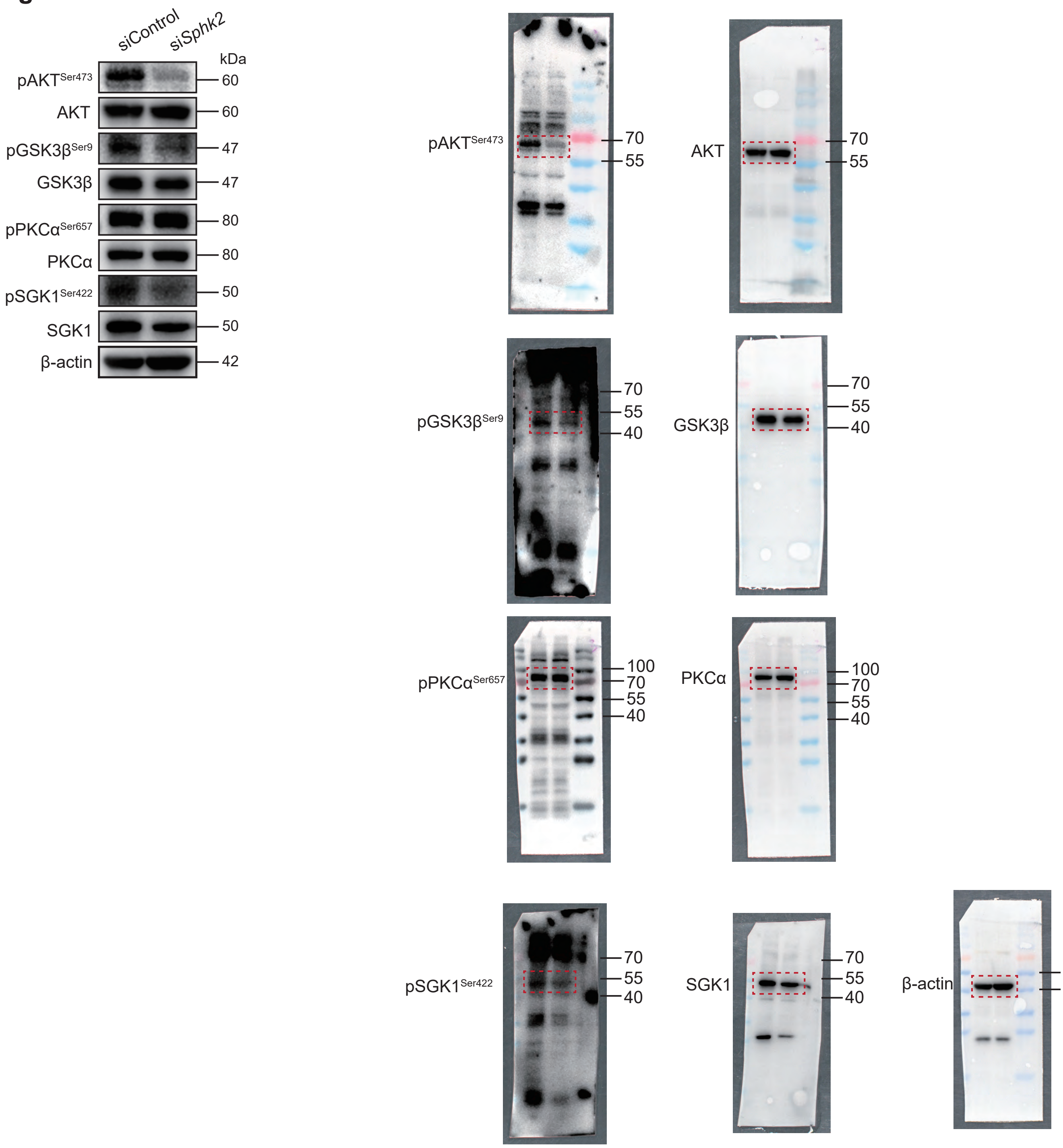
siControl
siSphk2
kDa
pAKTSer473
AKT
pGSK3βSer9
GSK3β
pPKCαSer657
PKCα
pSGK1Ser422
SGK1
β-actin
60
60
47
47
80
80
50
50
42
70
55
100
40
